# Red mark syndrome: Is the aquaculture water microbiome a keystone for understanding the disease aetiology?

**DOI:** 10.3389/fmicb.2023.1059127

**Published:** 2023-02-27

**Authors:** Antonia Bruno, Alessandra Cafiso, Anna Sandionigi, Andrea Galimberti, Davide Magnani, Amedeo Manfrin, Giulio Petroni, Maurizio Casiraghi, Chiara Bazzocchi

**Affiliations:** ^1^ZooPlantLab, Department of Biotechnologies and Biosciences, University of Milano-Bicocca, Milan, Italy; ^2^Department of Veterinary Medicine and Animal Science, University of Milan, Lodi, Italy; ^3^Quantia Consulting Srl, Milan, Italy; ^4^Experimental Zooprophylactic Institute of the Venezie (IZSVe), Legnaro, Italy; ^5^Department of Biology, University of Pisa, Pisa, Italy

**Keywords:** microbiome, infectious disease, red mark syndrome, built environment, water, rainbow trout, RMS-MLO

## Abstract

Aquaculture significantly contributes to the growing demand for food worldwide. However, diseases associated with intensive aquaculture conditions, especially the skin related syndromes, may have significant implications on fish health and industry. In farmed rainbow trout, red mark syndrome (RMS), which consists of multiple skin lesions, currently lacks recognized aetiological agents, and increased efforts are needed to elucidate the onset of these conditions. Most of the past studies were focused on analyzing skin lesions, but no study focused on water, a medium constantly interacting with fish. Indeed, water tanks are environmental niches colonized by microbial communities, which may be implicated in the onset of the disease. Here, we present the results of water and sediment microbiome analyses performed in an RMS-affected aquaculture facility, bringing new knowledge about the environmental microbiomes harbored under these conditions. On the whole, no significant differences in the bacterial community structure were reported in RMS-affected tanks compared to the RMS-free ones. However, we highlighted significant differences in microbiome composition when analyzing different samples source (*i.e.*, water and sediments). Looking at the finer scale, we measured significant changes in the relative abundances of specific taxa in RMS-affected tanks, especially when analyzing water samples. Our results provide worthwhile insight into a mostly uncharacterized ecological scenario, aiding future studies on the aquaculture built environment for disease prevention and monitoring.

## 1. Introduction

Aquaculture currently covers a primary role in facing the growing demand for the seafood market worldwide, providing a more sustainable approach to global fish production and high-quality proteins. Total fisheries and aquaculture production reached an all-time record of 214 million tons in 2020, comprising 178 million tons of aquatic animals. Global aquaculture production in 2020 reached a record 122.6 million tons, with 87.5 million tons of aquatic animals worth USD 264.8 billion (FAO, 2022). The increasing demand in the aquaculture sector is surpassing the ability of fisheries to keep up with the request for seafood (Rohani et al., 2022). For this reason, the introduction of new husbandry practices and technologies, and the intensification of well-established ones have been significantly incremented over the years. However, intensive aquaculture conditions and high stocking densities increase the risk of stressful conditions and can lead to compromised fish immune defences, generating direct and indirect economic implications, as observed, for example, in rainbow trout *Oncorhynchus mykiss* (Walbaum) (Bailey et al., 2020; Metselaar et al., 2022).

*Oncorhynchus mykiss* is currently the most abundant salmonid species employed in aquaculture worldwide (Crawford and Muir, 2008; Koutsikos et al., 2019), accounting for 97–98% of world trout production each year (EUMOFA, 2014). In the European Union, rainbow trout covers 64% of the value and 69% of the volume of total production in the freshwater segment (D’Agaro et al., 2022). In the context of intensive farming, reared rainbow trout can be subjected to several stressors and therefore be affected by several diseases (Lieke et al., 2020; Rohani et al., 2022), with the skin-related ones ranking first (Ellis et al., 2002; Oidtmann et al., 2013). Skin diseases in farmed rainbow trout are in continuous appearance, and many of them still lack an identified etiological agent (Oidtmann et al., 2013; Peeler et al., 2014). This is the case of red mark syndrome (RMS; also known as ‘cold water strawberry disease’ or ‘strawberry disease’ in the United States; Oidtmann et al., 2013; Metselaar et al., 2022), which consists of multiple skin lesions mostly found on the flanks of farmed, market-sized rainbow trout (over 100 g) and typically characterized by bright red, raised and non-ulcerative lesions between few millimeters to several centimeters in diameter (von Gersdorff Jørgensen et al., 2019; Metselaar et al., 2022). Despite the low mortality of the condition, RMS shows morbidity rates that can reach up to 90% of the farmed population (more frequently between 10 and 30%; Oidtmann et al., 2013), and the general appearance of the skin lesions can lead to significant product downgrading (Ferguson et al., 2006). Concerning the disease impact, a Danish report in 2016 reported that one third of the local trout farmers were dealing with the disease (von Gersdorff Jørgensen et al., 2019). These factors can cause significantly lowered incomes for farmers related to different levels of the supply chain, including table market (downgrade of the product) and restocking activities (Schmidt-Posthaus et al., 2009; von Gersdorff Jørgensen et al., 2019). However, currently, there are no reports on the actual economic impact of RMS in the fish industry (Metselaar et al., 2022).

In less than two decades, the disease has spread almost worldwide, with cases reported in several European countries, the Middle East, the Americas and Asia (Verner-Jeffreys et al., 2008; Kubilay et al., 2014; Sandoval et al., 2016; Oh et al., 2019a; Galeotti et al., 2021; Metselaar et al., 2022).

RMS’s morphological and histological features have been extensively described (Oidtmann et al., 2013), leading the disease to be clearly recognized. However, the etiological agent of RMS is still unknown, yet supposed to be bacterial-related, also supported by transmissibility studies (Schmidt et al., 2021). The transmission of RMS has indeed been described among affected and healthy individuals through direct and indirect contact (Schmidt et al., 2021; Orioles et al., 2022). For this reason, it cannot be excluded that effluents released by RMS-infected farms could reach natural water bodies and, in turn, provide pathogens exchange with wild populations. To date, RMS has been mainly reported in farmed rainbow trout (von Gersdorff Jørgensen et al., 2019). Similar lesions, but lacking in-depth histological and pathological investigations, have been sporadically observed in other freshwater species such as brown trout (*Salmo trutta*), cutthroat trout (*Oncorhynchus clarkii*), as well as wild-caught rainbow trout (Metselaar et al., 2022). Similar cases have also been reported in saltwater species, as sea bream and wild-caught common dab (*Limanda limanda*; Bruno et al., 2007; Vercauteren et al., 2020).

The most addressed agents associated with the disease are *Flavobacterium psychrophilum* and a *Rickettsia*-like organism (RLO; subsequently referred to as RMS-*Midichloria* like organism, RMS-MLO), a bacterium ascribed to the *Midichloriaceae* family (order *Rickettsiales*). However, the presence of *F. psychrophilum* has been reported in both RMS-affected and naïve fish (von Gersdorff Jørgensen et al., 2019; Metselaar et al., 2022), and no subsequent studies have found a clear association between this bacterium and the disease. On the contrary, a correlation between MLO presence in RMS lesions and their severity has been observed (Lloyd et al., 2008; Montagna et al., 2013; Cafiso et al., 2016; Schmidt et al., 2021).

Additionally, no specific RMS outbreak patterns have been reported except for the typical temperature ranges described in the literature (Oidtmann et al., 2013; Schmidt et al., 2021). Even in a single trout farm, the disease dissemination is not homogeneous in fish tanks nor repeated throughout the years (personal communication). Thus far, the majority of studies concerning RMS aetiology and pathology have been focused on histopathological, ultrastructural, or microbial evaluations directly linked to the fish (e.g., 16S rRNA gene libraries on the skin of affected/healthy individuals; Lloyd et al., 2008) or looking for specific targeted agents/vectors (Ferguson et al., 2006; Lloyd et al., 2008; Verner-Jeffreys et al., 2008; Cafiso et al., 2016; Pasqualetti et al., 2021; Metselaar et al., 2022). Investigations on the role of the farming environment in RMS are almost unexplored and mainly focused on management practices or facilities, diet, or water conditions (Ferguson et al., 2006; Lloyd et al., 2008; Verner-Jeffreys et al., 2008). Aquaculture systems represent fully-fledged built environments, harboring peculiar microbial communities (Gilbert and Stephens, 2018; Minich et al., 2021), and water is the primary compartment where pathogens should be controlled in aquaculture (De Schryver and Vadstein, 2014). Water tanks provide niches that can be colonized by different microbial taxa, which in turn form microbial communities that may change in composition, thus posing a risk to fish health conditions. In the aquatic environment, microorganisms and fish constantly interact, leading to favorable or disadvantageous effects for the vertebrate hosts depending on the microbial composition (Jahangiri et al., 2021). At the same time, the infectious ability of a single agent may be enhanced or diminished by the overall microbial community, thus affecting its virulence features (Teffer et al., 2022).

Rainbow trout are typically reared in flow-through and intensive aquaculture systems, which are supposed to be more prone to the proliferation of potential opportunistic microorganisms, as described for saltwater fish (Attramadal et al., 2012). So far, little is known about the microbial communities found in water and sediments of aquaculture environments in relation to healthy and diseased conditions in fish disorders.

Here we report, to our knowledge, the first investigation on the microbial communities of RMS-positive and RMS-negative rearing environments in a rainbow trout farm in natural conditions. RMS a etiology still needs to be unentangled, thus requiring any helpful hints to deepen the cause of the disease onset. From this perspective, evaluating the variation in the environmental microbial community in water could represent a starting point for future studies on specific potential etiological agents.

## 2. Materials and methods

### 2.1. Study site and sampling

**Figure 1:**
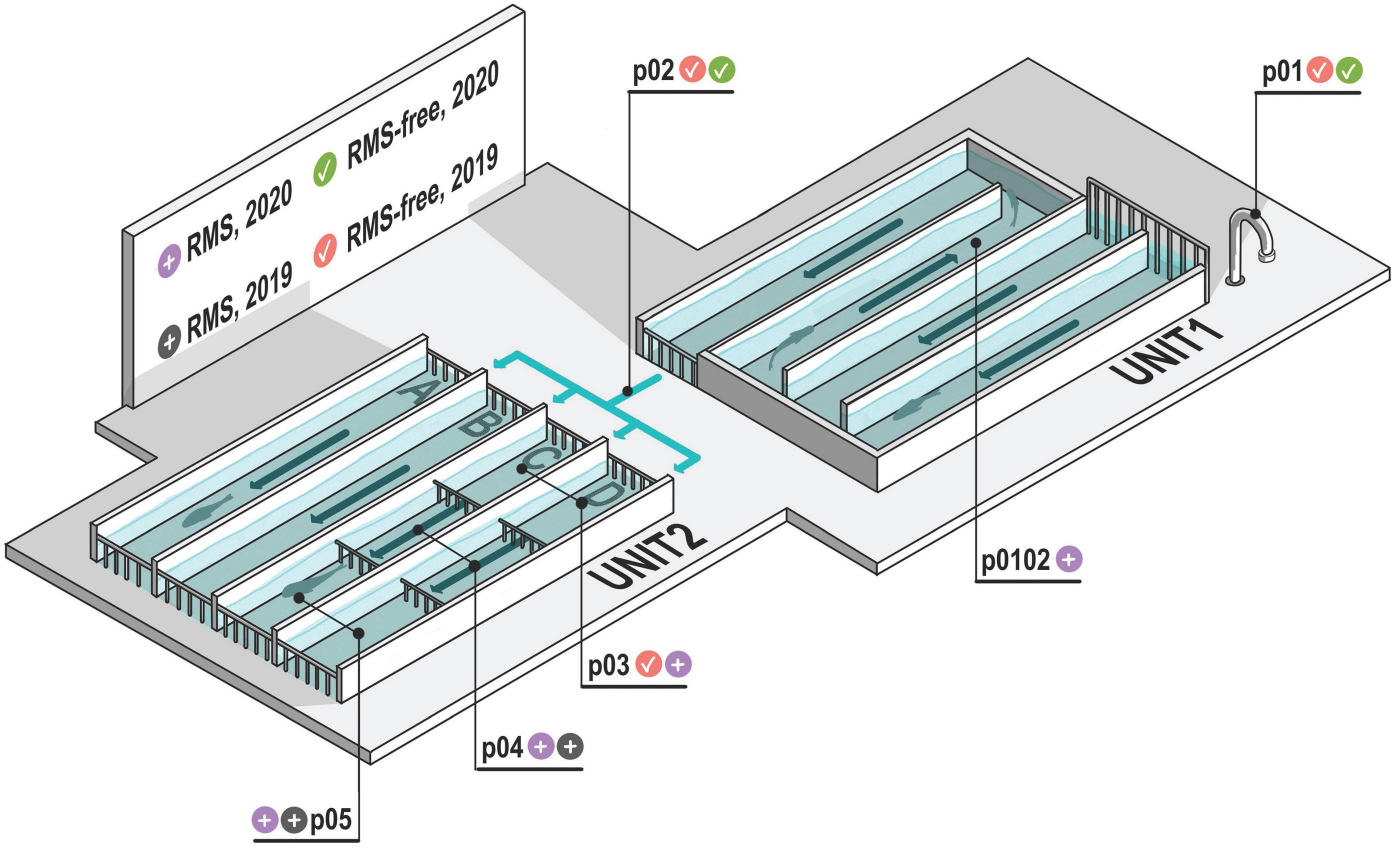
Schematic representation of the aquaculture plant examined. Sampling points (p01–p05) are represented. Sampling point p01 is bore well water which feeds the aquaculture system. Sampling point p02 is the water exiting Unit 1 and entering Unit 2. In p01 and p02 there are no fish. Sampling points where RMS was detected are indicated with gray and violet icons, considering the two sampling years (2019 and 2020, respectively). Similarly, sampling points where RMS was not detected (RMS-free) are indicated with red and green icons, considering the two sampling years (2019 and 2020, respectively). Arrows indicate the water flow.

A freshwater flow-through (FT) rainbow trout aquaculture in Storo (TN, Trentino Alto-Adige, NE Italy) was selected as the study site in two consecutive years. The fish farm was subjected to recurrent RMS outbreaks over the years, with random, not homogeneous tanks affected by the disease. The fish farm system was supplied by upstream 9°C bore well water, which is characterized by constant temperature and stable physico chemical characteristics, allowing comparable environmental conditions for both sampling years. The aquaculture system is organized into two main units (hereafter referred to as “Unit 1” and “Unit 2”), each one partitioned into four independent rows, which were in turn composed of three tanks, each delimited by separating grids. The volume of tanks in Unit 1 and 2 is 165 m^3^ and 225 m^3^, respectively. In the aquaculture units, temperature, dissolved oxygen levels, oxygen saturation and chemical parameters were constantly monitored. The general layout of the aquaculture system and the water flow are summarized in Figure 1.

The flow-through system is characterized by cemented tanks that are systematically cleaned every time a new fish batch is transferred inside. Fish density never exceeded 30 Kg/m^3^. Unit 1 was used for small-sized individuals (12–20 cm long), while fish up to the market size were reared in Unit 2. In detail, while the tanks of Unit 1 shared the same water, the four rows of Unit 2 (named, respectively, A, B, C, D) were independently supplied with the outlet water (p02) collected from Unit 1. The tanks of each row of Unit 2 were, respectively, named p03, p04, and p05, based on their distance from the inlet water channel.

To evaluate the possible influence of RMS outbreaks on the environmental microbial diversity, water and sediment of fish tanks were opportunistically sampled at two different sampling dates in concurrence with yearly RMS outbreaks (January 2019 and January 2020, hereafter “2019” and “2020”). Samples collected in sampling points where RMS was detected were labeled as “RMS.” Conversely, we used the label word “RMS-free” to identify those sampling points where no RMS was detected, including the sampling points (p01 and p02) where no fish were present. The sampling points, the sample sources and the presence/absence of RMS are summarized in Table 1. In detail, bore well water (p01), water and sediment of p02 and of the three tanks of C and D rows (p03-p05) were collected for both sampling dates. In addition, water and sediment from a tank in Unit 1 (p0102) were collected only in 2020 as positive for RMS. Sterile polypropylene sampling screw-cap bottles were used to collect 1 L of water. Sediment samples were collected from the bottom of tanks using a tight mesh net and then transferred in 50 ml Falcon tubes to collect 25 ml of sediment. Bottles and tubes were kept closed until the moment of sample collection. A total of 32 samples were collected. Samples were kept at 4°C and in the dark during transport and processed within 24 h.

**Table 1:** Samples list, grouped by “Condition_Souce” and detailed for “Sampling_point.”

Condition_Source	Sampling_point	Number of samples
Negative controls	NA	4
Negative controls Total		4
RMS-free_sediment	p02	4
	p03_C	2
	p03_D	1
RMS-free_sediment Total		7
RMS-free_water	p01	4
	p02	4
	p03_C	2
	p03_D	2
RMS-free_water Total		12
RMS_sediment	p0102	2
	p03_C	2
	p03_D	2
	p04_C	4
	p04_D	4
	p05_C	4
	p05_D	4
RMS_sediment Total		22
RMS_water	p0102	2
	p03_C	2
	p03_D	2
	p04_C	4
	p04_D	4
	p05_C	4
	p05_D	4
RMS_water Total		22
Sediment samples Total		29
Water samples Total		34
Grand Total		67

### 2.2. Sample processing and DNA extraction

One liter of water for each water sample was filtered using a vertical filtration apparatus and a vacuum pump (ME 2 NT vacuubrand). Filtration occurred in series, using nitrocellulose membrane filters of decreasing porosity to avoid filter clogging (pore size: 3, 0.45, 0.22 μm; diameter = 47 mm; membrane filter, Millipore, Burlington, Massachusetts, United States).

Tank sediments (25 ml of volume) were centrifuged, and the pellet and supernatant were processed separately. The supernatant was filtered using nitrocellulose membrane filters of 0.22 μm. Filters were stored at −80°C until DNA extraction.

After the processing, a total of 81 samples (51 from water, 15 from sediment supernatant, and 15 from sediment pellet samples) and 4 blanks (negative controls) were subjected to DNA extraction (Supplementary Table 1).

The filters were cut into smaller pieces with sterile instruments, and mechanical and chemical lysis were carried out as described in the protocol of DNeasy PowerWater Kit (Qiagen, Hilden, Germany) for water samples and DNeasy PowerSoil Kit (Qiagen, Hilden, Germany) for sediment (both pellet and supernatant) samples. One sediment replicate was lost during sample processing (p03D, 2019). To increase DNA yield, DNA was eluted in 75 μl of the elution buffer.

All the procedures were performed in a pre-amplification room under the flow cabinet, with sterilisation measures between samples using bleach and UV light.

### 2.3. Library preparation and high-throughput DNA sequencing

Bacterial DNA presence and abundance were measured by Quantitative Real-Time PCR (qPCR) for each of the 80 and 4 negative control samples, using the same primer pairs reported below for library preparation (without overhanging adapters). Briefly, qPCR assays were performed with AB7500 (Applied Biosystem) instrument. qPCR conditions included an initial denaturation at 95°C for 10 min, followed by 40 cycles of denaturation at 95°C for 15 s, and annealing-elongation at 55°C for 1 min. A final dissociation stage was performed. Amplification reaction (final volume 10 μl) consisted of 5.0 μl SsoFast EvaGreen Supermix with Low ROX (Bio-Rad, Hercules, CA), 0.1 μl each 10 μmoL l-1 primer solution, 2 μl DNA sample, and 2.8 μl of Milli-Q water. All samples and negative controls (no template) were run in triplicate. Threshold Cycles (Ct) values were converted into DNA counts/ml. A one-way analysis of variance ANOVA in combination with Tukey *post hoc* tests was used to find significant differences among groups in bacterial DNA concentration. A probability of *p* < 0.05 was considered to indicate a significant difference.

For library preparation, in the case of water samples, DNA extracts from membrane filters of similar porosity (0.45 μm and 0.22 μm) of the same sample were combined (50% volume each). All the remaining water samples were processed separately. In the case of sediment samples, DNA extracts from supernatant and pellet of the same sample were combined (50% volume each) and divided in to 2 replicates. Subsequently, starting from the obtained 67 samples (34 water and 29 sediment samples plus 4 negative controls; Table 1; Supplementary Table 1), the V3–V4 hypervariable regions of the 16S ribosomal DNA (rDNA) gene were amplified with S-D-Bact-0341-b-S-17, 5′-CCTACGGGNGGCWGCAG-3′ and S-D-Bact-0785-a-A-21, 5′-GACTACHVGGGTATCTAATCC-3′ primer pairs with overhanging adapters, according to the 16S Metagenomic Sequencing Library Preparation protocol (Illumina, SanDiego, CA, United States).

All the procedures were carried out in the laminar flow cabinet to avoid contamination with exogenous DNA and inter-sample contamination, and in separate rooms for the pre-and post-amplification steps, with dedicated personal protective equipment (PPE).

All libraries were sequenced on a MiSeq platform (Illumina, SanDiego, CA, United States) in two 2 × 300 bp paired-end runs by the Center for Omics Sciences at the IRCCS Ospedale San Raffaele (COSR, Milan, Italy).

### 2.4. Microbial composition and community structure analysis

The raw paired-end FASTQ reads were imported into the Quantitative Insights Into Microbial Ecology 2 program (QIIME2, ver. 2020.6; Caporaso et al., 2010; Bolyen et al., 2019) and demultiplexed native plugin. Raw reads were subsequently deposited into the European Nucleotide Archive (ENA; see Data Availability paragraph). The Divisive Amplicon Denoising Algorithm 2 (DADA2; Callahan et al., 2016) was used to quality filter, trim, denoise, and mergepairs the data. Chimeric sequences were removed using the consensus method. The taxonomic assignment of the amplicon sequence variants (ASVs) calculated was carried out using the feature-classifier2 plugin (Bokulich et al., 2018) implemented in QIIME2 against the SILVA SSU non-redundant database (138 release), adopting a consensus confidence threshold of 0.8.

Rarefaction curves were calculated to evaluate if the sequencing efforts generated enough data to well represent the overall microbial diversity in samples. The corresponding plot was generated with the phyloseq R package (McMurdie and Holmes, 2013).

We compared the samples considering the combination of sample source (water or sediment) and tank health condition (RMS-free or RMS), named in the manuscript as “Condition_Source” variable. In order to estimate the effect on alpha-diversity considering the variable ‘Condition_Source’, the Faith phylogenetic index (Faith PD) was calculated (Faith, 2016). The Kruskal–Wallis H test for all pairwise tests was used to compare the groups. We used Benjamini and Hochberg correction when multiple tests were applied, and the obtained q-value was reported.

Bray–Curtis dissimilarity was used to perform the community analyses (beta diversity), evenly sampled at 10,000 reads per sample, using the core-metrics-phylogenetic QIIME2 plugin. Samples with less than this threshold were excluded from the downstream analyses.

Statistical significance among groups (healthy water, healthy sediment, RMS water, RMS sediment) was determined by the ADONIS (permutation-based ANOVA, PerMANOVA) test (Anderson, 2005) with 999 permutations. PerMANOVA Pairwise contrast was performed, and the Benjamini-Hochberg FDR correction was used to calculate q-values. The test was performed using the beta-group-significance QIIME2 implemented plugin based on the adonis function in the vegan R package (Oksanen et al., 2007).

Thus, looking at a finer scale, a differential abundance analysis was carried out using the DESeq2 R package based on negative binomial generalized linear models (Love et al., 2014) to estimate differences between groups considering the relative abundance of ASVs assigned to the taxonomic rank of Genus. This data also generated a heatmap to detail the relative abundances of each sample analyzed.

### 2.5. qPCR for RMS-MLO

The presence of RMS-MLO was molecularly evaluated in DNA samples extracted from both water and sediment samples (*n* = 67, Table 1). qPCR was performed to amplify a 16S rDNA gene fragment of RMS-MLO using primers (16SrDNA-F: 5′-GCGGTTATCTGGGCAGTC-3′; 16SrDNA-R: 5′-TGCGACACCGAAACCTAAG-3′) and protocol previously described (Cafiso et al., 2016). A melting curve was determined with a transition rate of 0.5°C/s from 55°C to 95°C. Melting peaks were automatically calculated by iQ5 Optical System software (Bio-Rad, Hercules, CA, United States).

## 3. Results

### 3.1. Water parameters

In the aquaculture units, temperature, dissolved oxygen levels and oxygen saturation parameters were constantly monitored, ranging between 10 and 11°C, 9–10 mg/L, and 80–90%, respectively. Additional parameters measured during the year were pH = 7.5–8; total suspended solids <5 mg/L; NH_3_ < 0.02 mg/L; NH_4_ = 0.65 mg/L; NO_3_ = 1.2 mg/L; NO_2_ = 0.008 mg/L.

### 3.2. Sequence analysis

About 8,560,340 reads were obtained from 59 out of 67 samples: 4 negative controls and 4 samples (both replicas of p01 water 2019, p02 water 2019, and p03C water 2019) did not pass the library preparation or showed no reads after sequencing. After quality filtering, merging reads and chimaera removal of the two Illumina runs, we got 6,378,391 sequences, with a median frequency of 109,527 reads and the mean frequency of 103,392 reads per sample. We obtained 4,839 ASVs (Callahan et al., 2017; Supplementary Table 2).

### 3.3. Microbiome diversity composition and distribution

An exploratory analysis of bacterial 16S rDNA copies based on the qPCR results showed no significant differences in bacterial load considering different conditions (RMS vs. RMS-free) when comparing sediment samples. However, 16S rDNA copies resulted significantly lower in RMS-free water samples than in RMS water samples (*p* < 0.05; Supplementary Table 3; Supplementary Figure 1).

Rarefaction curves showed converging alpha diversity with increasing sequencing depth, confirming the suitability of the sampling effort (Supplementary Figure 2).

After rarefaction, 11 RMS-free and 44 RMS samples were obtained (Supplementary Table 2).

Microbiome biodiversity and composition were evaluated *via* alpha and beta diversity analyses. We compared the samples considering the combination of sample source (water or sediment) and tank condition (RMS-free or RMS; i.e., “Condition_Source” variable).

#### 3.3.1. Alpha diversity estimation

**Figure 2:**
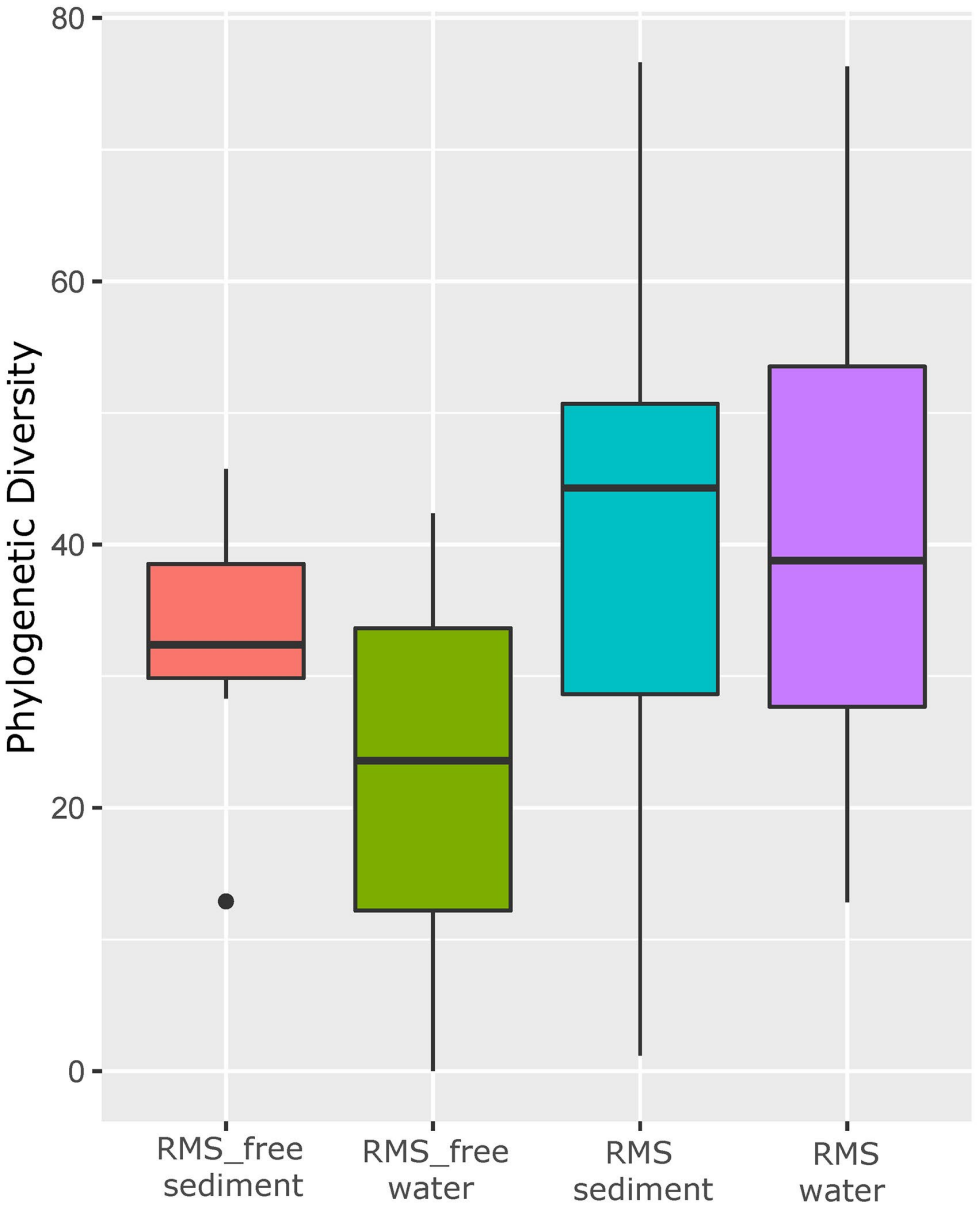
Alpha diversity based on Faith PD metric. Samples are grouped by “Condition_Source”, representing the combination of sample source (sediment or water) and tank condition (RMS-free or with fish affected by RMS).

Alpha diversity based on the Faith PD metric, which considers phylogenetic information, showed that water and sediment samples collected in tanks where RMS was detected had higher alpha diversity values (microbial diversity within the sample; Figure 2) than the other samples. The Kruskal–Wallis pairwise test was performed to compare alpha diversity values. Considering the different groups analyzed (RMS-free water, RMS-free sediment, RMS water, RMS sediment, considering the two sampling years), the Kruskal–Wallis H test based on Faith PD was significant (H = 9.3, *p* = 0.05). The alpha diversity measured by the Faith PD index changed significantly between the sediment where the presence of RMS was detected and the RMS-free sediment (H = 6.35, *p* = 0.01), with increasing microbial biodiversity in the RMS sediment. The same effect was observed between the RMS water and the RMS-free sediment (H = 4.1, *p* = 0.04). As regards the other combinations, no significant changes were observed (Supplementary Tables 4-6).

#### 3.3.2. Microbial composition

After the taxonomy assignment, we identified 37 bacterial Phyla, 93 Classes, 226 Orders, 362 Families, and 541 Genera (Supplementary Data 1).

**Figure 3:**
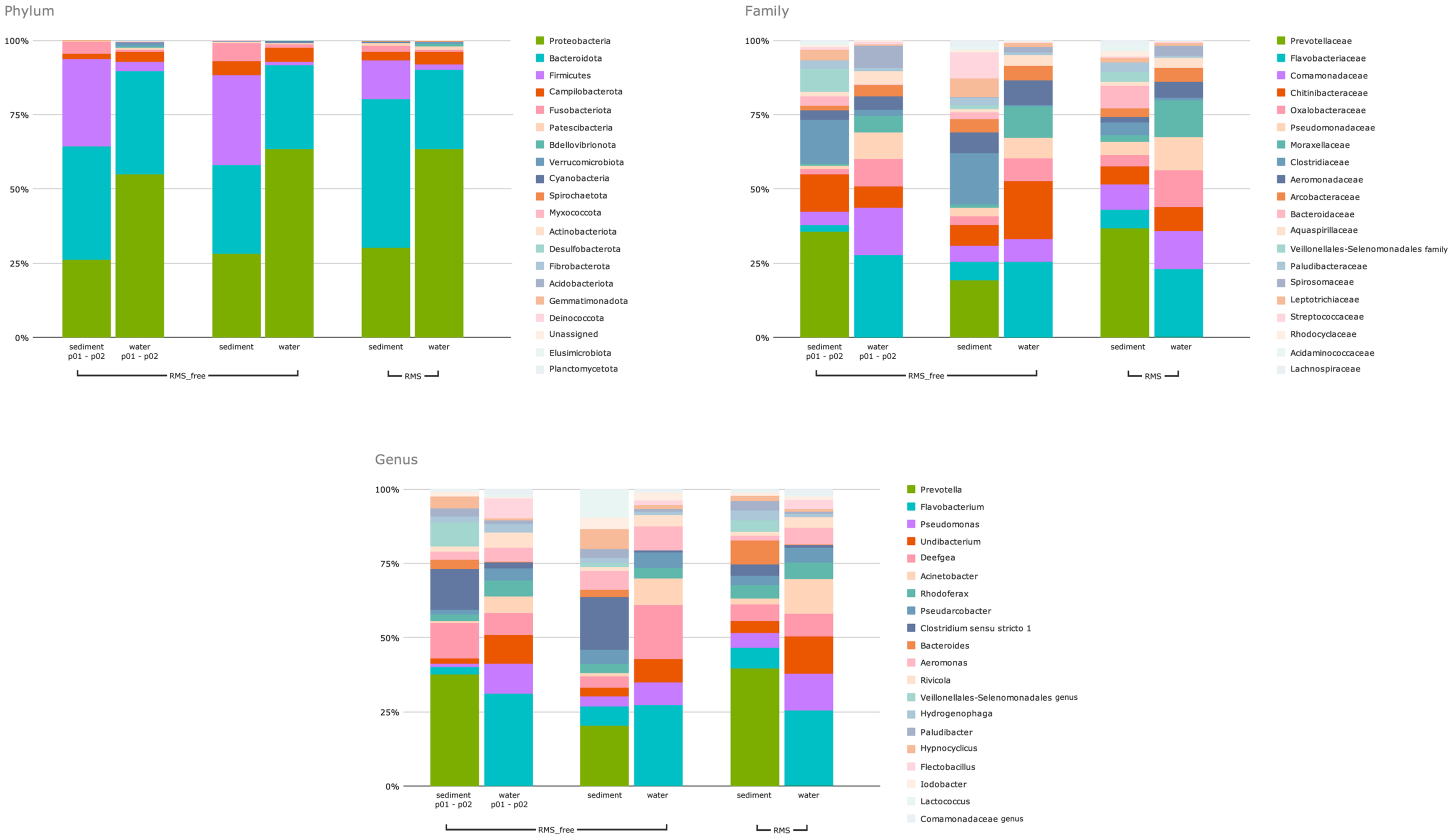
Bar chart regarding the distribution of the 20 most abundant Phyla, Families, and Genera.

Bar chart representation highlights the distribution of the 20 most abundant Phyla, Families, and Genera (Figure 3).

The relative abundance of the bacterial communities assigned at the taxonomic rank of Phylum showed that the samples were dominated by *Proteobacteria*, *Bacteroidota*, and *Firmicutes*, accounting for 93% of the total. Water samples showed a higher relative abundance of *Proteobacteria* than sediment samples. Conversely, sediment samples were characterized by a higher relative abundance of *Bacteroidota*.

In detail, *Proteobacteria* represented 26% of p02 RMS-free sediments, 28% of RMS-free sediments and 30% of RMS sediments. Conversely, they accounted for 55, 63, and 64% of p01 and p02 RMS-free water samples, RMS-free water samples, and RMS water samples, respectively. *Bacteroidota* were distributed as follows: 38, 30, and 27% in p01 and p02 RMS-free, RMS-free, and RMS sediment samples, respectively; 35, 28, and 27% in p01 and p02 RMS-free, RMS-free, and RMS water samples, respectively. The third most abundant phylum, *Firmicutes*, showed higher relative abundances in RMS-free sediment samples, also considering RMS-free p02 sampling points (30% in both cases), compared to RMS sediments (13%) and all the water samples tested (1–3%).

Overall, the most abundant families were *Prevotellaceae* (*Bacteroidota*), *Flavobacteriaceae* (*Bacteroidota*), *Comamonadaceae* (*Proteobacteria*), *Chitinibacteraceae* (*Proteobacteria*), *Oxalobacteraceae* (*Proteobacteria*), *Pseudomonadaceae* (*Proteobacteria*), *Moraxellaceae* (*Proteobacteria*), *Clostridiaceae* (*Firmicutes*), each falling within the range of 17–4% of the total sequences. Interestingly, differences were observed in the relative abundance of some microbial taxa considering the sample source. *Prevotellaceae* dominated sediment samples, while *Flavobacteriaceae* the water ones. Indeed, *Prevotellaceae* were detected at 31, 18, and 33% in p02 RMS-free sediments, RMS-free sediments, and RMS sediments, respectively. On the other hand, *Prevotellaceae* never exceeded 0.4% in water samples, regardless of the RMS condition. In contrast, sediment samples were composed of 2–6% of *Flavobacteriaceae*, whereas p01 and p02 RMS-free water samples, RMS-free water samples, and RMS water samples showed 25, 24, and 21% of *Flavobacteriaceae*, respectively.

Among the most abundant genera, in p01 and p02 RMS-free water samples (without fish) and RMS water samples, we found *Flavobacterium*, *Pseudomonas*, *Undibacterium*, *Acinetobacter*, and *Deefgea. Flavobacterium* in both RMS water and RMS-free water samples (21 and 25%, respectively, plus the 26% recovered in RMS-free p01 and p02). Noteworthy, the genus *Undibacterium* (*Oxalobacteraceae*) showed the highest relative abundance in water samples collected in tanks where RMS was detected (11%). *Prevotella* (*Prevotellaceae*) was dominant in both RMS sediment and RMS-free sediment samples (33 and 18%, respectively, without considering the 31% found in RMS-free p02).

Noteworthy, although we retrieved four ASVs affiliated with *Midichloriaceae*, none were related to RMS-MLO. DNA sequences assigned to the Order *Rickettsiales* were generally detected at a low percentage (<0.01%). A closer inspection revealed that the majority of them were either not resolved at a family/genus level or erroneously assigned as being either derived from mitochondria or affiliated to members of *Holosporales*, a clade previously included within *Rickettsiales* and now considered only distantly related to this group (Muñoz-Gómez et al., 2019).

We also reported the presence of *Candidatus* Megaira (*Rickettsiales*; *Rickettsiaceae*) at 0.009%, in 60% of RMS samples.

#### 3.3.3. Beta diversity analysis

**Figure 4:**
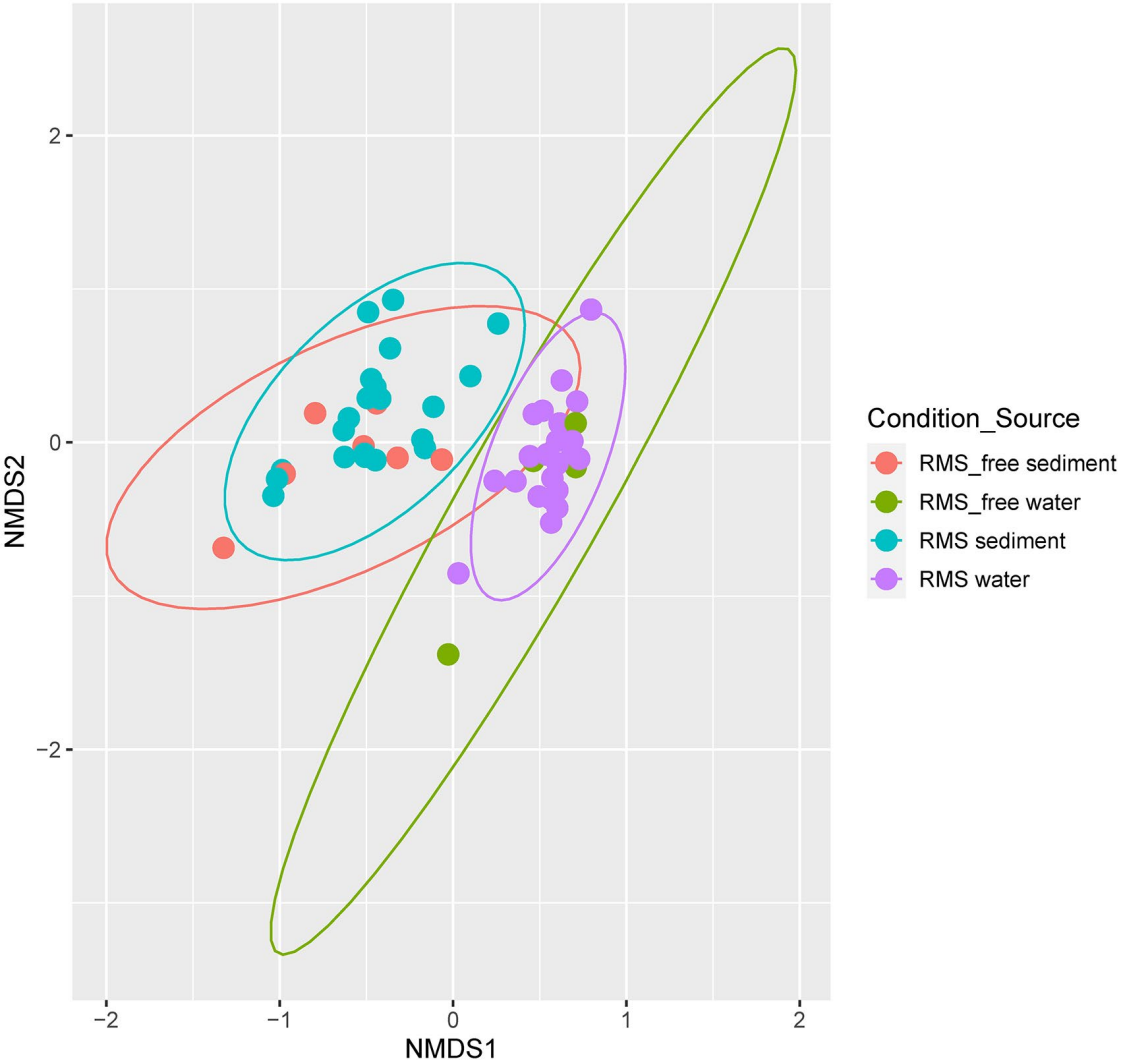
NMDS based on Bray–Curtis dissimilarity metric. Samples are colored by “Condition_Source”, representing the combination of sample source (sediment or water) and tank condition (RMS-free or RMS-affected).

To better explore the microbial differences among different sample sources (water and sediment) and different conditions (RMS-free and RMS), we computed beta diversity metrics (Bray-Curtis) and generated Non-metric Multidimensional Scaling (NMDS) plots. Samples clustered for samples source (water vs. sediment) and not for the condition (RMS-free vs. RMS; Figure 4).

Global PERMANOVA test was strongly significant for Condition_Source (*p* < 0.01), with the highest R2 value. All the combinations of Condition_Souce groups resulted in significant differences, except for comparing RMS-free water and RMS water. The comparison of RMS-free sediment vs. RMS sediment is near the value of significance (*p* = 0.089) but with a low R2 (=0.07; Supplementary Tables 7-8).

### 3.4. Differential abundances

In order to disentangle any contribution of RMS-free or RMS condition at a finer scale, a differential abundance analysis was carried out using the negative binomial generalized linear models. Significant relative abundance differences were computed considering the two different sample sources, sediment and water.

**Figure 5:**
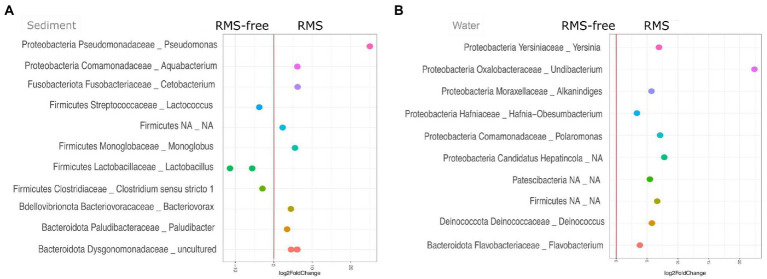
Sediment **(A)** and water **(B)** samples, comparing the conditions RMS vs. RMS-free, assigned at the taxonomic rank of Genus. Abundances are expressed as log2 of fold change.

When looking at sediment samples, we noticed that *Lactococcus* (*Streptococcaceae*), *Lactobacillus* (*Lactobacillaceae*), and *Clostridium sensu stricto* 1 (*Clostridiaceae*) genera were significantly lower in RMS samples than in the RMS-free ones. Conversely, *Paludibacter* (*Paludibacteraceae*), *Bacteriovorax* (*Bacteriovoraceae*), *Monoglobus* (Monoglobaceae), *Aquabacterium*, an uncultured bacterium belonging to *Dysgomonadaceae* family, *Cetobacterium*, and *Pseudomonas* were significantly more abundant in sediment samples collected in the RMS condition than RMS-free tanks (Figure 5A).

On the other hand, considering water samples, the genera *Undibacterium* (*Oxalobacteraceae*), *Polaromonas* (*Comamonadaceae*), *Yersinia* (*Yersiniaceae*), *Deinococcus* (*Deinococcaceae*), *Alkanindiges* (*Moraxellaceae*), *Flavobacterium* (*Flavobacteriaceae*), *Hafnia-Obesumbacterium* (*Hafniaceae*), an unidentified genus belonging to “*Candidatus* Hepatincola,” and an unidentified genus belonging to *Patescibacteria* were significantly more abundant in water samples collected in tanks where RMS was detected. No taxon was significantly less abundant in water samples from RMS sampling points (Figure 5B).

**Figure 6:**
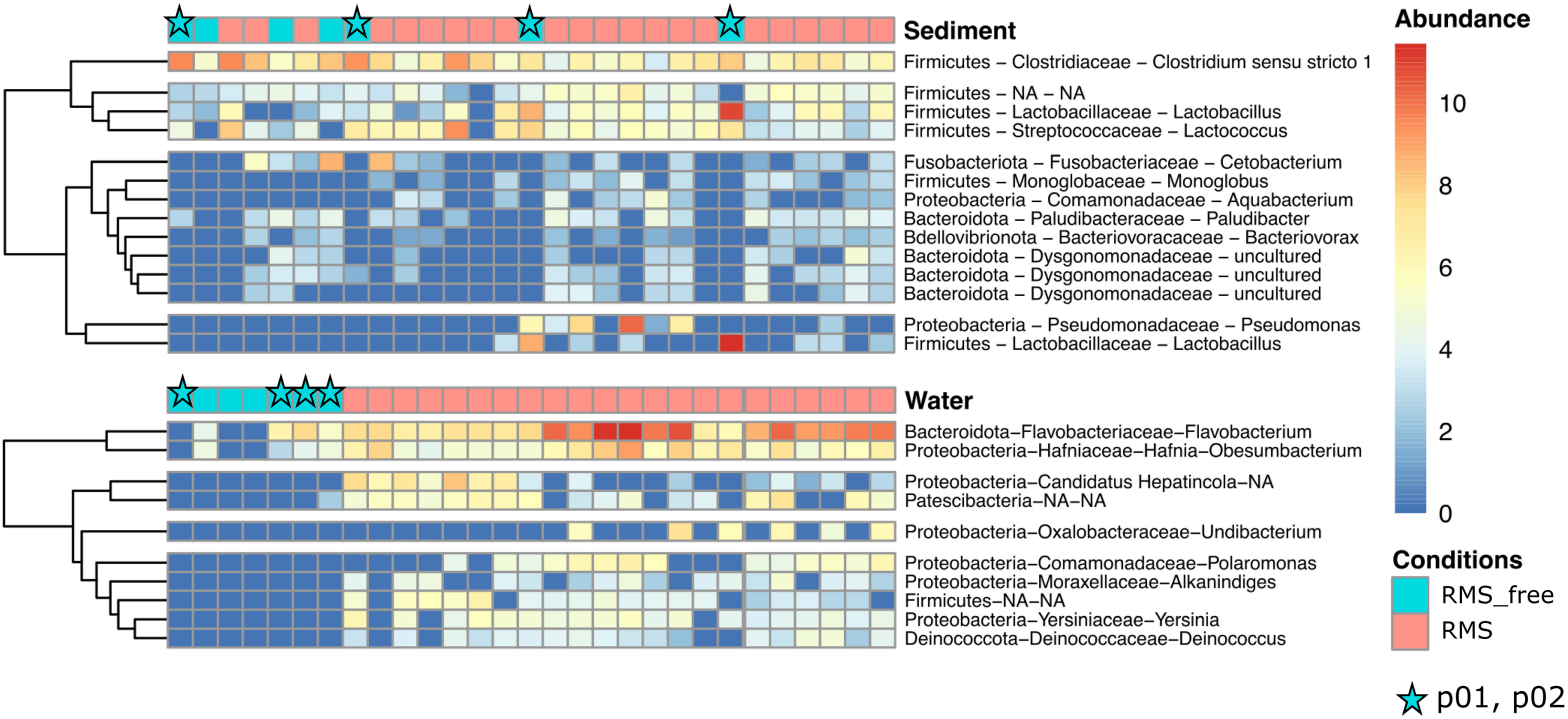
Heatmaps showing the relative abundances of ASVs, assigned at the taxonomic rank of Genus, for water and sediment samples, comparing the conditions RMS vs. RMS-free. Abundances are expressed as log2 of fold change. Stars indicate samples collected in p01 and p02 (no fish presence).

A heatmap was generated for both the sediment and the water samples to detail the taxonomic contribution for each sample (Figure 6). In particular, we observed a more homogenous distribution of abundances in water samples compared to the sediment ones. Indeed, when looking at the Condition (RMS-free vs. RMS), each water sample collected at RMS sites showed a similar abundance pattern for all the taxa and each taxon was found enriched in almost all RMS water samples. In particular, the first cluster composed of two taxa assigned to *Hafnia-Obesumbacterium* and *Flavobacterium* showed a marked enrichment of these taxa in all the RMS samples.

Conversely, sediment samples appeared to be more inhomogeneous in terms of relative abundances of taxa considering the Condition. Indeed, most taxa showed positive fold change in relative abundances in some RMS samples but not in others, and *vice versa* in some RMS-free samples.

### 3.5. RMS-MLO quantitative real time PCR results

Since no member of the *Midichloriaceae* family was detected looking at the data obtained by 16S V3-V4 HTS, a qPCR approach was performed to evaluate the presence of RMS-MLO bacteria in both water and sediment of RMS-free and RMS conditions. The threshold cycles of the obtained amplifications were below the lower limit of the dynamic range of the reaction. For this reason, the qPCR approach was only used to assess the presence/absence of RMS-MLO based on the evaluation of melting curves of positive samples compared to those of positive controls used in each qPCR reaction. The results of the molecular analyses for RMS-MLO presence are reported in Supplementary Table 9. RMS-MLO was generally detected in both water and sediment where RMS occurred, while water and sediments of RMS-free tanks resulted negative for the presence of RMS-MLO. However, in one water and three sediment samples of RMS tanks, RMS-MLO DNA was not detected.

Additionally, the sediment of p02 was positive for the presence of RMS-MLO in 2019.

## 4. Discussion

The aquaculture environment is paramount for fish health, as it can be the source and the vector of potential pathogens. Understanding the relationship between the built environment of tanks and fish health may be necessary for disentangling disease aetiology and predicting disease outbreaks. Moreover, to date, only limited studies are available concerning the comparative microbial communities in aquaculture environments where diseases are occurring (Infante-Villamil et al., 2021).

Red mark syndrome currently lacks recognized aetiological agents. In our study, we had the opportunity to collect samples of water and sediments from an FT aquaculture system with an RMS outbreak. Given the fact that a pathogen is supposed to be the cause of the disease and the water and aquaculture environment could serve as vectors, investigating water and sediment microbiomes may be crucial. Our study provides the first report of the diversity of microbial communities harbored by a freshwater FT aquaculture system, in which the presence of fish affected by RMS was ascertained.

### 4.1. Whole microbial diversity in an aquaculture ecosystem where RMS is detected

On the whole, we measured a striking microbial diversity in water and sediment samples, accounting for thousands of ASVs and spanning among nearly 40 bacterial Phyla and about one hundred Classes.

This aspect is not surprising since aquatic ecosystems biodiversity is well documented (Tanentzap et al., 2019; Garner et al., 2020; Scolari et al., 2021; Mills et al., 2022), especially in groundwater and bore water ecosystems (Bautista-De Los Santos et al., 2016; Bruno et al., 2017, 2018, 2021; Kaestli et al., 2019).

In addition, the beta diversity analyses revealed significant differences in microbial composition between sediment and water samples, irrespective of the condition of RMS-free or RMS tanks. Recent studies well documented that sample source or sample type can be the major driver of microbial composition (Ma et al., 2020), also when focusing on aquaculture plant ecosystems (Minich et al., 2020; Minich et al., 2021; Testerman et al., 2022). Noteworthy, in our case-study, the substantial similarity in the microbial composition of samples belonging to the same sample sources was confirmed in the two consecutive years of samplings.

However, alpha diversity estimation showed that water and sediment samples collected in tanks where RMS was detected had higher values compared to the RMS-free samples.

If in general diversity is considered a good parameter to estimate ecosystem health, contrasting results in terms of alpha diversity have been reported in microbial communities harbored by aquatic ecosystems when healthy and diseased status are compared. Indeed, if the study of Xiong et al. (2015) in farmed shrimps found that OTU richness and phylogenetic diversity were higher in the healthy water environment, other researchers reported no changes in alpha diversity (Zhang et al., 2014; Li et al., 2017; Zheng et al., 2017). It is clear that systematic and broad analyses of aquaculture-associated microbiomes are lacking, impeding an exhaustive and correct estimation of the microbial dynamics in healthy and diseased status (Infante-Villamil et al., 2021).

### 4.2. Microbiome composition of RMS-free and RMS samples

When we tracked the microbiome of water samples from the natural source (bore water) to the outflow through the tanks, comparing RMS-free and RMS samples, we found that the overall water microbiome was similar in composition, despite the animal presence and/or to the RMS diagnosis. Indeed, the top 10 genera in water samples were almost shared, which is consistent with other studies investigating freshwater and aquaculture ecosystems (Testerman et al., 2022). The only exception was represented by *Acidovorax*, a denitrifying bacterium commonly found in water ecosystems (Xu et al., 2022), retrieved mainly in bore water. However, some of the most abundant genera detected deserve a note for monitoring applications. As an example, *Acinetobacter* presence should be monitored carefully, since some species can be pathogens in aquaculture, transmitting antibiotic resistance genes. Fish mortality caused by several *Acinetobacter* species, such as *A. baumannii*, *A. lwoffii*, *A. johnsonii*, and *A. calcoaceticus*, has been well documented in rainbow trout (Kozińska et al., 2014). Similarly, *Pseudomonas* species are opportunistic bacteria, resistant to the betalactam group of antibiotics, and responsible for septicemic diseases among freshwater fish (De Kievit et al., 2001; Oh et al., 2019b). However, we must consider that not all the species belonging to these genera are harmful, but the natural presence of water ecosystems.

Similar conclusions can be drawn from sediment samples, even if they clearly showed a shift in composition compared to their complementary water samples. In this case, *Prevotella* dominated all the sediment samples, probably deriving from fish gut debris. In general, bacteria of the genus *Prevotella* are known for their ability to degrade complex plant polysaccharides; indeed, in humans, these bacteria have been clearly associated with plant-based diets, which are rich in fibers (Chen et al., 2017).

To deepen the investigation, we searched for the taxa that significantly varied, considering relative abundance, in RMS-free or RMS conditions. Our data showed that subtle but significant differences exist in RMS and RMS-free sampling points, both for sediment and water samples. Noteworthy, the distribution of taxa in water appeared to be more homogeneous than in sediment: after DeSeq analysis the resulting taxa in water all showed an increase in RMS condition, whereas sediment samples did not. If water is mixed by the flow, sediment accumulates over time, probably in an uneven manner. Moreover, if sediment represents the “memory” of the tank, water spreads in real time host-associated and environment-associated bacteria.

The specific taxa identified were common environmental bacteria (such as *Patescibateria*, *Aquabacterium*, *Polaromonas*) and host-associated bacteria released in the tanks (such as *Lactococcus* and *Lactobacillus*), and some of the genera detected included opportunistic pathogens (such as *Pseudomonas* and *Clostridium sensu stricto* I). The genus *Undibacterium*, significantly more abundant in water samples collected in tanks where RMS was detected, is commonly found in freshwater, soil, and fish, but its pathogenicity remains unclear (Kim et al., 2014; Kämpfer et al., 2016; Lee et al., 2019). Similarly, *Flavobacteria* are commonly found in aquatic environments and their isolation in freshwater is correlated with aquaculture activities (Singh et al., 2021). In the present study, *Flavobacteria* were abundant in the water compartment of both RMS-free and RMS tanks. The reason could be addressed to the presence of fish, since this bacterial taxon is generally isolated from the skin and gills of the aquatic host (Singh et al., 2021; Terova et al., 2021). Nevertheless, together with *Hafnia-Obesumbacterium,* the genus *Flavobacterium* showed a significant enrichment in all the RMS water samples. In general, both genera include pathogenic or innoxius species (Loch and Faisal, 2015; Nguyen et al., 2021; Foysal et al., 2022), impeding a direct implication with the disease. However, this unique and consistent distribution homogeneity can hint at the development of monitoring strategies for preventing or managing the disease.

A significant decrease of specific taxa belonging to the phylum *Firmicutes*, typically found in aquaculture sediments (Dai et al., 2021), was observed in RMS tanks compared to RMS-free ones: lactic acid bacteria (LAB), namely *Lactobacillus* and *Lactococcus* (Heikkinen et al., 2006; Desai et al., 2012), were significantly lower in sediment samples of tanks housing RMS fish compared to RMS-free tanks. LAB normally occur in fish gastrointestinal (GI) tract, and in *O. mykiss* they have been suggested to exert a probiotic effect by hindering the growth, adherence and colonization of potentially harmful bacteria in the digestive tract (Huong et al., 2014; Ringø et al., 2018). We can not exclude that RMS could have influenced the gut microbial community in the affected fish leading to a decrease of LAB, resulting in a modified bacterial population found in the organic matter in tank sediments. No abnormalities are usually detected in organs other than spleen in RMS-affected fish (Galeotti et al., 2021; Orioles et al., 2022). However, necropsies performed on RMS-affected individuals in Italy have highlighted occasional enteritis and gross gut alterations (e.g., swollen intestine and intestinal muscle inflammation; A. Cafiso personal communication; Oidtmann et al., 2013). Disorders and stressful conditions can lead to altered feeding behavior and dysfunctions of the GI system, potentially leading to dysbiosis of the gut (Ringø et al., 2014; Parshukov et al., 2019). The decreased abundance of LAB in diseased *O. mykiss* is in line with previous results from studies focused on evaluating the differential *O. mykiss* gut microbiota based on the infection status (Parshukov et al., 2019). RMS might have additionally affected the presence of bacteria ascribed to *Clostridium sensu stricto* clade in the GI microbiome of the RMS-affected fish leading, in turn, to a reduced presence in sediments. Further studies focused on investigating the microbial community of RMS-affected and healthy *O. mykiss* could support these results. The differences observed between RMS-free and RMS sediment in the rearing tanks could thus reflect the GI health status of the fish population.

### 4.3. A focus on *Rickettsiales* and RMS-MLO

In the past 20 years, the number of endosymbionts described within the bacterial order *Rickettsiales* has constantly grown and a critical revision of the group has been proposed. Among the taxa differentially enriched in water samples where RMS was detected, we found DNA sequences assigned to *Candidatus* Hepatincola. *Candidatus* Hepatincola was first described as a symbiont of a terrestrial isopod (Wang et al., 2004) and attributed to the order *Rickettsiales*. Recent studies also documented its presence associated with other arthropods and, interestingly, it was never described in aquaculture ecosystems. The phylogeny of this candidate genus is discussed and it was recently proposed a new family, *Candidatus* Hepatincolaceae, referring to a new proposed order, *Holosporales* nov. ord. (Szokoli et al., 2016). Studies on terrestrial isopods suggested that *Candidatus* Hepatincola is extracellular (Wang et al., 2004), horizontally transmitted (Wang et al., 2007) and may have a specific role in host trophic adaptation (Delhoumi et al., 2020).

Concerning *Rickettsiales*, only a few members could be identified to the genus/family.

Among them, it is worth mentioning four ASV clustering within *Midichloriaceae,* all associated with genera “*Candidatus* Bandiella” (Senra et al., 2016) / “*Candidatus* Aquarickettsia” (Klinges et al., 2019; the two genera are entangled/not properly resolved). “*Candidatus* Bandiella” has been frequently reported as an intracellular symbiont of freshwater ciliate protists from the genus *Euplotes* (Boscaro et al., 2019) and its role in the interaction with the host has not been clarified yet. On the contrary, “*Candidatus* Aquarickettsia” has been originally reported in association with marine invertebrates, *i.e.*, corals (Klinges et al., 2019) and its presence correlates with host susceptibility to diseases (Klinges et al., 2019). In addition, this bacterium is horizontally transmitted (Baker et al., 2022) and increases in phosphate-rich environments (Klinges et al., 2019).

The only true *Rickettsiales* member detected in the present study using HTS was *Candidatus* Megaira, an intracellular bacterium associated with a wide span of hosts in ecological contexts ranging from freshwater to marine systems (Lanzoni et al., 2019).

The presence of RMS-MLO DNA, assessed through qPCR analysis, was detected in those tanks where RMS-affected fish was present. This observation is in line with previous studies showing a correlation between RMS-MLO and skin lesions of RMS-affected fish (Lloyd et al., 2008; Cafiso et al., 2016; Schmidt et al., 2021; Orioles et al., 2022). However, scant bacterial loads were observed in the analyzed matrixes, as indicated by Ct values exceeding the lower limit of the qPCR reaction dynamic range. This result could explain the absence of HTS reads ascribable to members of the *Midichloriaceae* family, although *in silico* PCR analyses showed complete matching of the primers targeting the V3–V4 hypervariable regions of the RMS-MLO 16S rDNA gene sequence. Nevertheless, the scarce bacterial load could additionally support the negative qPCR results obtained in one water and three sediment samples of tanks housing RMS-affected fish. Interestingly, RMS-MLO DNA was detected in the sediment of water exiting Unit 1 and entering Unit 2 (p02). Despite the lack of any fish presence in Unit 1, it could not be excluded that RMS-MLO hosted/vectored by putative vectors or temporarily free RMS-MLO in the extracellular environment (Castelli et al., 2019) could somehow have reached the water tank (Unit 1). The bacterium, likely hosted by a putative vector as previously hypothesized elsewhere (e.g., protozoans; Pasqualetti et al., 2021), could thus have been occasionally included in the organic matter deposited in between Unit 1 and Unit 2.

## 5. Conclusion

The current study has contributed new knowledge concerning the composition of microbial communities in flow-through aquaculture systems when the presence of RMS is reported. Due to the unknown aetiology of this emerging disease, a complete picture of the factors that may be involved in RMS outbreak and spread is needed. Among these factors, the water-associated microbiome has always been neglected.

Although risks arising from the presence of pathogens also need further investigation, our results lay a foundation for the design of aquaculture systems that can be less prone to the emergence and spread of fish diseases. Water can serve as a vector and its microbial ecology can have a role in the modulation of disease outbreak and spread, and fish response.

The novel insights obtained in this study not only add relevant information about the context of the disease, but also they could put RMS under the spotlight and increase interest in research related to this disease. In the future, studies should be performed including host-associated microbial communities in healthy and RMS-affected rainbow trout, looking at the aquaculture built environment on the whole.

## Data availability statement

The data presented in the study are deposited in the European Nucleotide Archive (ENA) under accession number PRJEB56297.

## Author contributions

AB, AS, AC, and CB conceived and designed the experiments. AB, AC, and DM performed the experiments. AS, AB, and GP analyzed the data. AB, AS, CB, AG, MC, GP, and AC drafted the manuscript and figures. All authors contributed to the article and approved the submitted version.

## Funding

The research reported in this publication was partially supported by PRIN_MIUR 2012 (code 2012A4F828) to CB.

## Conflict of interest

AS was employed by Quantia Consulting Srl.

The remaining authors declare that the research was conducted in the absence of any commercial or financial relationships that could be construed as a potential conflict of interest.

## Publisher’s note

All claims expressed in this article are solely those of the authors and do not necessarily represent those of their affiliated organizations, or those of the publisher, the editors and the reviewers. Any product that may be evaluated in this article, or claim that may be made by its manufacturer, is not guaranteed or endorsed by the publisher.

## References

[ref100] Anderson M. J.. (2005). PERMANOVA: A FORTRAN Computer Program for Permutational Multivariate Analysis of Variance. Auckland: University of Auckland.

[ref1] Attramadal K. J. Salvesen I. Xue R. Øie G. Størseth T. R. Vadstein O.. (2012). Recirculation as a possible microbial control strategy in the production of marine larvae. Aquac. Eng. 46, 27–39. doi: 10.1016/j.aquaeng.2011.10.003

[ref2] Bailey C. Holland J. W. Secombes C. J. Tafalla C. (2020). A portrait of the immune response to proliferative kidney disease (PKD) in rainbow trout. Parasite Immunol. 42:e12730. doi: 10.1111/pim.12730, PMID: 32403171 PMC7507176

[ref3] Baker L. J. Reich H. G. Kitchen S. A. Grace Klinges J. Koch H. R. Baums I. B.. (2022). The coral symbiont *Candidatus* Aquarickettsia is variably abundant in threatened Caribbean acroporids and transmitted horizontally. ISME J. 16, 400–411. doi: 10.1038/s41396-021-01077-8, PMID: 34363004 PMC8776821

[ref4] Bautista-De Los Santos Q. M. Schroeder J. L. Sevillano-Rivera M. C. Sungthong R. Ijaz U. Z. Sloan W. T.. (2016). Emerging investigators series: microbial communities in full-scale drinking water distribution systems–a meta-analysis. Environ. Sci. 2, 631–644. doi: 10.1039/C6EW00030D

[ref5] Bokulich N. A. Kaehler B. D. Rideout J. R. Dillon M. Bolyen E. Knight R.. (2018). Optimizing taxonomic classification of marker-gene amplicon sequences with QIIME 2’s q2-feature-classifier plugin. Microbiome 6, 1–17. doi: 10.1186/s40168-018-0470-z 29773078 PMC5956843

[ref6] Bolyen E. Rideout J. R. Dillon M. R. Bokulich N. A. Abnet C. C. Al-Ghalith G. A.. (2019). Reproducible, interactive, scalable and extensible microbiome data science using QIIME 2. Nat. Biotechnol. 37, 852–857. doi: 10.1038/s41587-019-0209-9, PMID: 31341288 PMC7015180

[ref7] Boscaro V. Husnik F. Vannini C. Keeling P. J. (2019). Symbionts of the ciliate *Euplotes*: diversity, patterns and potential as models for bacteria–eukaryote endosymbioses. Proc. R. Soc. B 286:20190693. doi: 10.1098/rspb.2019.0693, PMID: 31311477 PMC6661354

[ref8] Bruno D. Crumlish M. LaPatra S. Noguera P. Verner-Jeffreys D. (2007). Workshop on salmonid skin diseases. European Association of Fish Pathologists.

[ref9] Bruno A. Sandionigi A. Bernasconi M. Panio A. Labra M. Casiraghi M. (2018). Changes in the drinking water microbiome: effects of water treatments along the flow of two drinking water treatment plants in a urbanized area, Milan (Italy). Front. Microbiol. 9:2557. doi: 10.3389/fmicb.2018.02557, PMID: 30429832 PMC6220058

[ref10] Bruno A. Sandionigi A. Magnani D. Bernasconi M. Pannuzzo B. Consolandi C.. (2021). Different effects of mineral versus vegetal granular activated carbon filters on the microbial community composition of a drinking water treatment plant. Front. Ecol. Evol. 9:615513. doi: 10.3389/fevo.2021.615513

[ref11] Bruno A. Sandionigi A. Rizzi E. Bernasconi M. Vicario S. Galimberti A.. (2017). Exploring the under-investigated “microbial dark matter” of drinking water treatment plants. Sci. Rep. 7, 1–7. doi: 10.1038/srep44350 28290543 PMC5349567

[ref12] Cafiso A. Sassera D. Serra V. Bandi C. McCarthy U. Bazzocchi C. (2016). Molecular evidence for a bacterium of the family *Midichloriaceae* (order *Rickettsiales*) in skin and organs of the rainbow trout *Oncorhynchus mykiss* (Walbaum) affected by red mark syndrome. J. Fish Dis. 39, 497–501. doi: 10.1111/jfd.12371, PMID: 25828398

[ref13] Callahan B. J. McMurdie P. J. Holmes S. P. (2017). Exact sequence variants should replace operational taxonomic units in marker-gene data analysis. ISME J. 11, 2639–2643. doi: 10.1038/ismej.2017.119, PMID: 28731476 PMC5702726

[ref14] Callahan B. J. McMurdie P. J. Rosen M. J. Han A. W. Johnson A. J. A. Holmes S. P. (2016). DADA2: high-resolution sample inference from Illumina amplicon data. Nat. Methods 13, 581–583. doi: 10.1038/nmeth.3869, PMID: 27214047 PMC4927377

[ref15] Caporaso J. G. Kuczynski J. Stombaugh J. Bittinger K. Bushman F. D. Costello E. K.. (2010). QIIME allows analysis of high-throughput community sequencing data. Nat. Methods 7, 335–336. doi: 10.1038/nmeth.f.303, PMID: 20383131 PMC3156573

[ref16] Castelli M. Sabaneyeva E. Lanzoni O. Lebedeva N. Floriano A. M. Gaiarsa S.. (2019). *Deianiraea*, an extracellular bacterium associated with the ciliate *Paramecium*, suggests an alternative scenario for the evolution of *Rickettsiales*. ISME J. 13, 2280–2294. doi: 10.1038/s41396-019-0433-9, PMID: 31073215 PMC6776064

[ref17] Chen T. Long W. Zhang C. Liu S. Zhao L. Hamaker B. R. (2017). Fiber-utilizing capacity varies in *Prevotella*-versus *Bacteroides*-dominated gut microbiota. Sci. Rep. 7, 1–7. doi: 10.1038/s41598-017-02995-4 28572676 PMC5453967

[ref18] Crawford S. S. Muir A. M. (2008). Global introductions of salmon and trout in the genus *Oncorhynchus:* 1870–2007. Rev. Fish Biol. Fish. 18, 313–344. doi: 10.1007/s11160-007-9079-1

[ref19] D’Agaro E. Gibertoni P. Esposito S. (2022). Recent trends and economic aspects in the rainbow trout (*Oncorhynchus mykiss*) sector. Appl. Sci. 12:8773. doi: 10.3390/app12178773

[ref20] Dai L. Liu C. Peng L. Song C. Li X. Tao L.. (2021). Different distribution patterns of microorganisms between aquaculture pond sediment and water. J. Microbiol. 59, 376–388. doi: 10.1007/s12275-021-0635-5, PMID: 33630250

[ref21] De Kievit T. R. Parkins M. D. Gillis R. J. Srikumar R. Ceri H. Poole K.. (2001). Multidrug efflux pumps: expression patterns and contribution to antibiotic resistance in *Pseudomonas aeruginosa* biofilms. Antimicrob. Agents Chemother. 45, 1761–1770. doi: 10.1128/AAC.45.6.1761-1770.2001, PMID: 11353623 PMC90543

[ref22] De Schryver P. Vadstein O. (2014). Ecological theory as a foundation to control pathogenic invasion in aquaculture. ISME J. 8, 2360–2368. doi: 10.1038/ismej.2014.84, PMID: 24892581 PMC4260705

[ref23] Delhoumi M. Catania V. Zaabar W. Tolone M. Quatrini P. Achouri M. (2020). The gut microbiota structure of the terrestrial isopod *Porcellionides pruinosus* (Isopoda: Oniscidea). Eur. Zool. J. 87, 357–368. doi: 10.1080/24750263.2020.1781269

[ref24] Desai A. R. Links M. G. Collins S. A. Mansfield G. S. Drew M. D. Van Kessel A. G.. (2012). Effects of plant-based diets on the distal gut microbiome of rainbow trout (*Oncorhynchus mykiss*). Aquaculture 350-353, 134–142. doi: 10.1016/j.aquaculture.2012.04.005

[ref25] Ellis T. North B. Scott A. P. Bromage N. r., Porter, M., and Gadd, D. (2002). The relationships between stocking density and welfare in farmed rainbow trout. J. Fish Biol. 61, 493–531. doi: 10.1111/j.1095-8649.2002.tb00893.x

[ref26] EUMOFA (2014). European market observatory for fisheries and aquaculture products. 2014 edition: the EU fish market.

[ref27] Faith D. P. (2016). “The PD phylogenetic diversity framework: linking evolutionary history to feature diversity for biodiversity conservation” in Biodiversity conservation and phylogenetic systematics, eds R. Pellens and P. Grandcolas (Cham: Springer), 39–56.

[ref28] FAO (2022). The state of world fisheries and aquaculture 2022. Towards Blue Transformation. Rome, FAO.

[ref29] Ferguson H. W. Girons A. Rizgalla G. LaPatra S. Branson E. J. MacKenzie K.. (2006). Strawberry disease in rainbow trout in Scotland: pathology and association with *Flavobacterium* psychrophilum. Vet. Rec. 158, 630–632. doi: 10.1136/vr.158.18.630, PMID: 16679482

[ref30] Foysal M. J. Dao T. T. T. Fotedar R. Gupta S. K. Tay A. Chaklader M. R. (2022). Sources of protein diet differentially stimulate the gut and water microbiota under freshwater crayfish, marron (Cherax cainii, Austin 2002) culture. Environ. Microbiol. Rep. 14, 286–298. doi: 10.1111/1758-2229.13049, PMID: 35130581 PMC9303337

[ref31] Galeotti M. Volpatti D. Byadgi O. Beraldo P. Orioles M. Sarti M.. (2021). Red mark syndrome (RMS) in farmed rainbow trout: first report of outbreaks in Bosnia and Herzegovina. J. Fish Dis. 44, 627–631. doi: 10.1111/jfd.13336, PMID: 33476400

[ref32] Garner R. E. Gregory-Eaves I. Walsh D. A. (2020). Sediment metagenomes as time capsules of lake microbiomes. mSphere 5, e00512–e00520. doi: 10.1128/mSphere.00512-20 33148818 PMC7643826

[ref33] Gilbert J. A. Stephens B. (2018). Microbiology of the built environment. Nat. Rev. Microbiol. 16, 661–670. doi: 10.1038/s41579-018-0065-5 30127345

[ref34] Heikkinen J. Vielma J. Kemiläinen O. Tiirola M. Eskelinen P. Kiuru T.. (2006). Effects of soybean meal based diet on growth performance, gut histopathology and intestinal microbiota of juvenile rainbow trout (*Oncorhynchus mykiss*). Aquaculture 261, 259–268. doi: 10.1016/j.aquaculture.2006.07.012

[ref35] Huong N. T. Hien L. T. Wenresti G. G. Hai N. T. (2014). Bacterial population in intensive tilapia (*Oreochromis niloticus*) culture pond sediment in Hai Duong province, Vietnam. Int. J. Fish. Aquac. 6, 133–139. doi: 10.5897/IJFA14.0425

[ref36] Infante-Villamil S. Huerlimann R. Jerry D. R. (2021). Microbiome diversity and dysbiosis in aquaculture. Rev. Aquac. 13, 1077–1096. doi: 10.1111/raq.12513

[ref37] Jahangiri L. Shinn A. P. Pratoomyot J. Bastos Gomes G. (2021). Unveiling associations between ciliate parasites and bacterial microbiomes under warm-water fish farm conditions – a review. Rev. Aquac. 13, 1097–1118. doi: 10.1111/raq.12514

[ref38] Kaestli M. O’Donnell M. Rose A. Webb J. R. Mayo M. Currie B. J.. (2019). Opportunistic pathogens and large microbial diversity detected in source-to-distribution drinking water of three remote communities in Northern Australia. PLoS Negl. Trop. Dis. 13:e0007672. doi: 10.1371/journal.pntd.0007672, PMID: 31487283 PMC6728021

[ref39] Kämpfer P. Irgang R. Busse H. J. Poblete-Morales M. Kleinhagauer T. Glaeser S. P.. (2016). *Undibacterium danionis* sp. nov. isolated from a zebrafish (*Danio rerio*). Int. J. Syst. Evol. Microbiol. 66, 3625–3631. doi: 10.1099/ijsem.0.001244, PMID: 27307140

[ref40] Kim S. J. Moon J. Y. Weon H. Y. Hong S. B. Seok S. J. Kwon S. W. (2014). *Undibacterium jejuense* sp. nov. and *Undibacterium seohonense* sp. nov., isolated from soil and freshwater, respectively. Int. J. Syst. Evol. Microbiol. 64, 236–241. doi: 10.1099/ijs.0.056846-0, PMID: 24048872

[ref41] Klinges J. G. Rosales S. M. McMinds R. Shaver E. C. Shantz A. A. Peters E. C.. (2019). Phylogenetic, genomic, and biogeographic characterization of a novel and ubiquitous marine invertebrate-associated *Rickettsiales* parasite, *Candidatus* Aquarickettsia rohweri, gen. nov., sp. nov. ISME J. 13, 2938–2953. doi: 10.1038/s41396-019-0482-0, PMID: 31384012 PMC6863919

[ref42] Koutsikos N. Vardakas L. Zogaris S. Perdikaris C. Kalantzi O.-I. Economou A. N. (2019). Does rainbow trout justify its high rank among alien invasive species? Insights from a nationwide survey in Greece. Aquat. Conserv. Mar. Freshwat. Ecosyst. 29, 409–423. doi: 10.1002/aqc.3025

[ref43] Kozińska A. Paździor E. Pękala A. Niemczuk W. (2014). *Acinetobacter johnsonii* and *Acinetobacter lwoffii*-the emerging fish pathogens. J. Vet. Res. 58, 193–199. doi: 10.2478/bvip-2014-0029

[ref44] Kubilay A. Ciftci S. Yildirim P. Didinen B. I. Metin S. Demirkan T.. (2014). First observation of red mark syndrome (RMS) in cultured rainbow trout (*Oncorhynchus mykiss* Walbaum, 1792) in Turkey. Bull. Eur. Assoc. Fish Pathol. 34, 95–101.

[ref45] Lanzoni O. Sabaneyeva E. Modeo L. Castelli M. Lebedeva N. Verni F.. (2019). Diversity and environmental distribution of the cosmopolitan endosymbiont *Candidatus* Megaira. Sci. Rep. 9:1179. doi: 10.1038/s41598-018-37629-w, PMID: 30718604 PMC6362216

[ref46] Lee S. Y. Kang W. Kim P. S. Kim H. S. Sung H. Shin N. R.. (2019). *Undibacterium piscinae* sp. nov., isolated from Korean shiner intestine. Int. J. Syst. Evol. Microbiol. 69, 3148–3154. doi: 10.1099/ijsem.0.003604, PMID: 31385778

[ref130] Li H. Zeng J. Ren L. Wang J. Xing P. Wu Q. L. (2017). Contrasting patterns of diversity of abundant and rare bacterioplankton in freshwater lakes along an elevation gradient. Limnol. Oceanogr 62, 1570–1585. doi: 10.1002/lno.10518

[ref47] Lieke T. Meinelt T. Hoseinifar S. H. Pan B. Straus D. L. Steinberg C. E. W. (2020). Sustainable aquaculture requires environmental-friendly treatment strategies for fish diseases. Rev. Aquac. 12, 943–965. doi: 10.1111/raq.12365

[ref48] Lloyd S. J. LaPatra S. E. Snekvik K. R. St-Hilaire S. Cain K. D. Call D. R. (2008). Strawberry disease lesions in rainbow trout from southern Idaho are associated with DNA from a *Rickettsia*-like organism. Dis. Aquat. Org. 82, 111–118. doi: 10.3354/dao01969, PMID: 19149374

[ref49] Loch T. P. Faisal M. (2015). Emerging flavobacterial infections in fish: a review. J. Adv. Res. 6, 283–300. doi: 10.1016/j.jare.2014.10.009, PMID: 26257926 PMC4522593

[ref120] Love M. I. Huber W. Anders S. (2014). Moderated estimation of fold change and dispersion for RNA-seq data with DESeq2. Genome Biol. 15(12), 1–21. doi: 10.1186/s13059-014-0550-8, PMID: 25516281 PMC4302049

[ref50] Ma B. Wang Y. Ye S. Liu S. Stirling E. Gilbert J. A.. (2020). Earth microbial co-occurrence network reveals interconnection pattern across microbiomes. Microbiome 8, 1–12. doi: 10.1186/s40168-020-00857-2 32498714 PMC7273686

[ref51] McMurdie P. J. Holmes S. (2013). phyloseq: an R package for reproducible interactive analysis and graphics of microbiome census data. PLoS One 8:e61217. doi: 10.1371/journal.pone.0061217, PMID: 23630581 PMC3632530

[ref52] Metselaar M. Orioles M. Galeotti M. Adams A. Thompson K. D. (2022). Red mark syndrome – current state of knowledge. Aquaculture 549:737748. doi: 10.1016/j.aquaculture.2021.737748

[ref53] Mills M. Lee S. Mollenkopf D. Wittum T. Sullivan S. M. P. Lee J. (2022). Comparison of environmental microbiomes in an antibiotic resistance-polluted urban river highlights periphyton and fish gut communities as reservoirs of concern. Sci. Total Environ. 851:158042. doi: 10.1016/j.scitotenv.2022.158042, PMID: 35973543

[ref54] Minich J. J. Nowak B. Elizur A. Knight R. Fielder S. Allen E. E. (2021). Impacts of the marine hatchery built environment, water and feed on mucosal microbiome colonization across ontogeny in Yellowtail Kingfish, Seriola lalandi. Front. Mar. Sci. 8:676731. doi: 10.3389/fmars.2021.676731, PMID: 36248701 PMC9563383

[ref55] Minich J. J. Poore G. D. Jantawongsri K. Johnston C. Bowie K. Bowman J.. (2020). Microbial ecology of Atlantic salmon (*Salmo salar*) hatcheries: impacts of the built environment on fish mucosal microbiota. Appl. Environ. Microbiol. 86, e00411–e00420. doi: 10.1128/AEM.00411-20 32303543 PMC7267192

[ref56] Montagna M. Sassera D. Epis S. Bazzocchi C. Vannini C. Lo N.. (2013). “*Candidatus* Midichloriaceae” fam. nov.(*Rickettsiales*), an ecologically widespread clade of intracellular alphaproteobacteria. Appl. Environ. Microbiol. 79, 3241–3248. doi: 10.1128/AEM.03971-12, PMID: 23503305 PMC3685259

[ref58] Muñoz-Gómez S. A. Hess S. Burger G. Lang B. F. Susko E. Slamovits C. H.. (2019). An updated phylogeny of the *Alphaproteobacteria* reveals that the parasitic *Rickettsiales* and *Holosporales* have independent origins. elife 8, p.e42535. doi: 10.7554/eLife.42535, PMID: 30789345 PMC6447387

[ref59] Nguyen T. T. T. Foysal M. J. Fotedar R. Gupta S. K. Siddik M. A. Tay C.-Y. (2021). The effect of two dietary protein sources on water quality and the aquatic microbial communities in marron (*Cherax cainii*) culture. Microb. Ecol. 82, 299–308. doi: 10.1007/s00248-021-01681-3, PMID: 33432372

[ref60] Oh W. T. Giri S. S. Yun S. Kim H. J. Kim S. G. Kim S. W.. (2019a). Emergence of rickettsial infection in rainbow trout (*Oncorhynchus mykiss*) fry displaying the appearance of red mark syndrome in Korea. Microorganisms 7:302. doi: 10.3390/microorganisms7090302, PMID: 31470673 PMC6780055

[ref61] Oh W. T. Kim J. H. Jun J. W. Giri S. S. Yun S. Kim H. J.. (2019b). Genetic characterization and pathological analysis of a novel bacterial pathogen, *Pseudomonas tructae*, in rainbow trout (*Oncorhynchus mykiss*). Microorganisms 7:432. doi: 10.3390/microorganisms7100432, PMID: 31658660 PMC6843698

[ref62] Oidtmann B. Lapatra S. E. Verner-Jeffreys D. Pond M. Peeler E. J. Noguera P. A.. (2013). Differential characterization of emerging skin diseases of rainbow trout--a standardized approach to capturing disease characteristics and development of case definitions. J. Fish Dis. 36, 921–937. doi: 10.1111/jfd.12086 23448696

[ref63] Oksanen J. Kindt R. Legendre P. O’Hara B. Stevens M. H. H. Oksanen M. J.. (2007). The vegan package. Community ecology package 10, 719.

[ref64] Orioles M. Saccà E. Metselaar M. Bulfoni M. Cesselli D. Galeotti M. (2022). Observations on red mark syndrome in juvenile rainbow trout farmed in RAS system. J. Fish Dis. 45, 1889–1892. doi: 10.1111/jfd.13707, PMID: 35964248 PMC9804602

[ref65] Parshukov A. Kashinskaya E. Simonov E. Hlunov O. Izvekova G. Andree K.. (2019). Variations of the intestinal gut microbiota of farmed rainbow trout, *Oncorhynchus mykiss* (Walbaum), depending on the infection status of the fish. J. Appl. Microbiol. 127, 379–395. doi: 10.1111/jam.14302, PMID: 31066161

[ref66] Pasqualetti C. Schmidt J. G. Cafiso A. Gammuto L. Lanzoni O. Sepulveda D.. (2021). Double trouble: could *Ichthyophthirius multifiliis* be a vehicle for the bacterium associated with red mark syndrome in rainbow trout, *Oncorhynchus mykiss*? Aquaculture 533:736230. doi: 10.1016/j.aquaculture.2020.736230

[ref67] Peeler E. J. Ryder D. Thrush M. A. Mewett J. Hulland J. Feist S. W. (2014). Acute dermatitis in farmed trout: an emerging disease. J. Fish Dis. 37, 1021–1029. doi: 10.1111/jfd.12241, PMID: 24720525

[ref68] Ringø E. Hoseinifar S. H. Ghosh K. Doan H. V. Beck B. R. Song S. K. (2018). Lactic acid bacteria in finfish—an update. Front. Microbiol. 9:1818. doi: 10.3389/fmicb.2018.01818, PMID: 30147679 PMC6096003

[ref69] Ringø E. ZhiGang Z. Suxu H. Olsen R. E. (2014). Effect of stress on intestinal microbiota of Arctic charr, Atlantic salmon, rainbow trout and Atlantic cod: a review. Afr. J. Microbiol. Res. 8, 609–618. doi: 10.5897/AJMR2013.6395

[ref70] Rohani M. F. Islam S. M. Hossain M. K. Ferdous Z. Siddik M. A. B. Nuruzzaman M.. (2022). Probiotics, prebiotics and synbiotics improved the functionality of aquafeed: upgrading growth, reproduction, immunity and disease resistance in fish. Fish Shellfish Immunol. 120, 569–589. doi: 10.1016/j.fsi.2021.12.037, PMID: 34963656

[ref71] Sandoval C. Infante J. Abad J. Ferguson H. W. Paredes E. Valdebenito S.. (2016). Case report: strawberry disease in farmed Chilean rainbow trout. J. Aquat. Anim. Health 28, 1–10. doi: 10.1080/08997659.2015.1114534, PMID: 26913369

[ref72] Schmidt J. G. Henriksen N. H. Olesen N. J. (2021). Antibiotic treatment alleviates red mark syndrome symptoms in rainbow trout (*Oncorhynchus mykiss*) and reduces load of *Midichloria*-like organism. Aquaculture 532:736008. doi: 10.1016/j.aquaculture.2020.736008

[ref73] Schmidt-Posthaus H. Bergmann W. Knüsel R. Heistinger H. Licek E. (2009). Appearance of red mark syndrome/cold water strawberry disease in Switzerland and Austria. Dis. Aquat. Org. 88, 65–68. doi: 10.3354/dao02152, PMID: 20183966

[ref74] Scolari F. Sandionigi A. Carlassara M. Bruno A. Casiraghi M. Bonizzoni M. (2021). Exploring changes in the microbiota of *Aedes albopictus:* comparison among breeding site water, larvae, and adults. Front. Microbiol. 12:624170. doi: 10.3389/fmicb.2021.624170, PMID: 33584626 PMC7876458

[ref75] Senra M. V. Dias R. J. Castelli M. Silva-Neto I. D. Verni F. Soares C. A.. (2016). A house for two—double bacterial infection in *Euplotes woodruffi* Sq1 (Ciliophora, Euplotia) sampled in Southeastern Brazil. Microb. Ecol. 71, 505–517. doi: 10.1007/s00248-015-0668-6, PMID: 26381539

[ref76] Singh S. Mallik S. K. Kala K. Shahi N. Pathak R. Giri A. K.. (2021). Characterization of *Flavobacterium columnare* from farmed infected rainbow trout, *Oncorhynchus mykiss* (Walbaum, 1792) of central Indian Himalayan region, India. Aquaculture 544:737118. doi: 10.1016/j.aquaculture.2021.737118

[ref78] Szokoli F. Sabaneyeva E. Castelli M. Krenek S. Schrallhammer M. Soares C. A. G.. (2016). “*Candidatus* Fokinia solitaria”, a novel “stand-alone” symbiotic lineage of *Midichloriaceae (Rickettsiales*). PLoS One 11:e0145743. doi: 10.1371/journal.pone.0145743, PMID: 26731731 PMC4701390

[ref79] Tanentzap A. J. Fitch A. Orland C. Emilson E. J. Yakimovich K. M. Osterholz H.. (2019). Chemical and microbial diversity covary in fresh water to influence ecosystem functioning. Proc. Natl. Acad. Sci. 116, 24689–24695. doi: 10.1073/pnas.1904896116, PMID: 31740592 PMC6900631

[ref80] Teffer A. K. Hinch S. G. Miller K. M. Patterson D. A. Bass A. L. Cooke S. J.. (2022). Host-pathogen-environment interactions predict survival outcomes of adult sockeye salmon (*Oncorhynchus nerka*) released from fisheries. Mol. Ecol. 31, 134–160. doi: 10.1111/mec.16214, PMID: 34614262

[ref81] Terova G. Gini E. Gasco L. Moroni F. Antonini M. Rimoldi S. (2021). Effects of full replacement of dietary fishmeal with insect meal from *Tenebrio molitor* on rainbow trout gut and skin microbiota. J. Anim. Sci. Biotechnol. 12, 1–14. doi: 10.1186/s40104-021-00551-9 33536078 PMC7860006

[ref82] Testerman T. Beka L. McClure E. A. Reichley S. R. King S. Welch T. J.. (2022). Detecting Flavobacterial fish pathogens in the environment via high-throughput community analysis. Appl. Environ. Microbiol. 88, e02092–e02021. doi: 10.1128/AEM.02092-21 34788066 PMC8788675

[ref83] Vercauteren M. Decostere A. Chiers K. (2020). First report of lesions resembling red mark syndrome observed in wild-caught common dab (*Limanda limanda*). J. Fish Dis. 43, 147–151. doi: 10.1111/jfd.13104, PMID: 31724198

[ref84] Verner-Jeffreys D. Pond M. Peeler E. Rimmer G. Oidtmann B. Way K.. (2008). Emergence of cold water strawberry disease of rainbow trout *Oncorynchus mykiss* in England and Wales: outbreak investigations and transmission studies. Dis. Aquat. Org. 79, 207–218. doi: 10.3354/dao01916, PMID: 18589997

[ref85] von Gersdorff Jørgensen L. Schmidt J. G. Chen D. Kania P. W. Buchmann K. Olesen N. J. (2019). Skin immune response of rainbow trout (*Oncorhynchus mykiss*) experimentally exposed to the disease red mark syndrome. Vet. Immunol. Immunopathol. 211, 25–34. doi: 10.1016/j.vetimm.2019.03.008, PMID: 31084890

[ref86] Wang Y. Brune A. Zimmer M. (2007). Bacterial symbionts in the hepatopancreas of isopods: diversity and environmental transmission. FEMS Microbiol. Ecol. 61, 141–152. doi: 10.1111/j.1574-6941.2007.00329.x, PMID: 17506824

[ref87] Wang Y. Stingl U. Anton-Erxleben F. Zimmer M. Brune A. (2004). ‘*Candidatus* Hepatincola porcellionum’gen. nov., sp. nov., a new, stalk-forming lineage of *Rickettsiales* colonizing the midgut glands of a terrestrial isopod. Arch. Microbiol. 181, 299–304. doi: 10.1007/s00203-004-0655-7, PMID: 14770277

[ref88] Xiong J. Wang K. Wu J. Qiuqian L. Yang K. Qian Y.. (2015). Changes in intestinal bacterial communities are closely associated with shrimp disease severity. Appl. Microbiol. Biotechnol. 99, 6911–6919. doi: 10.1007/s00253-015-6632-z, PMID: 25947250

[ref89] Xu B. He J. Zou H. Zhang J. Deng L. Yang M.. (2022). Different responses of representative denitrifying bacterial strains to gatifloxacin exposure in simulated groundwater denitrification environment. Sci. Total Environ. 850:157929. doi: 10.1016/j.scitotenv.2022.157929, PMID: 35952894

[ref90] Zhang D. Wang X. Xiong J. Zhu J. Wang Y. Zhao Q.. (2014). Bacterioplankton assemblages as biological indicators of shrimp health status. Ecol. Indic. 38, 218–224. doi: 10.1016/j.ecolind.2013.11.002

[ref91] Zheng Y. Yu M. Liu J. Qiao Y. Wang L. Li Z.. (2017). Bacterial community associated with healthy and diseased Pacific white shrimp (*Litopenaeus vannamei*) larvae and rearing water across different growth stages. Front. Microbiol. 8:1362. doi: 10.3389/fmicb.2017.01362, PMID: 28769916 PMC5513922

