# Supplementary material for: Red mark syndrome: Is the aquaculture water microbiome a keystone for understanding the disease aetiology?
Source: Front Microbiol. 2023 Feb 27;14:1059127. doi: 10.3389/fmicb.2023.1059127 (PMC10010170; doi:10.3389/fmicb.2023.1059127)
Supplement: Supplementary file 2 [file Data_Sheet_2.DOCX]

***Supplementary Material***

## Supplementary Figures


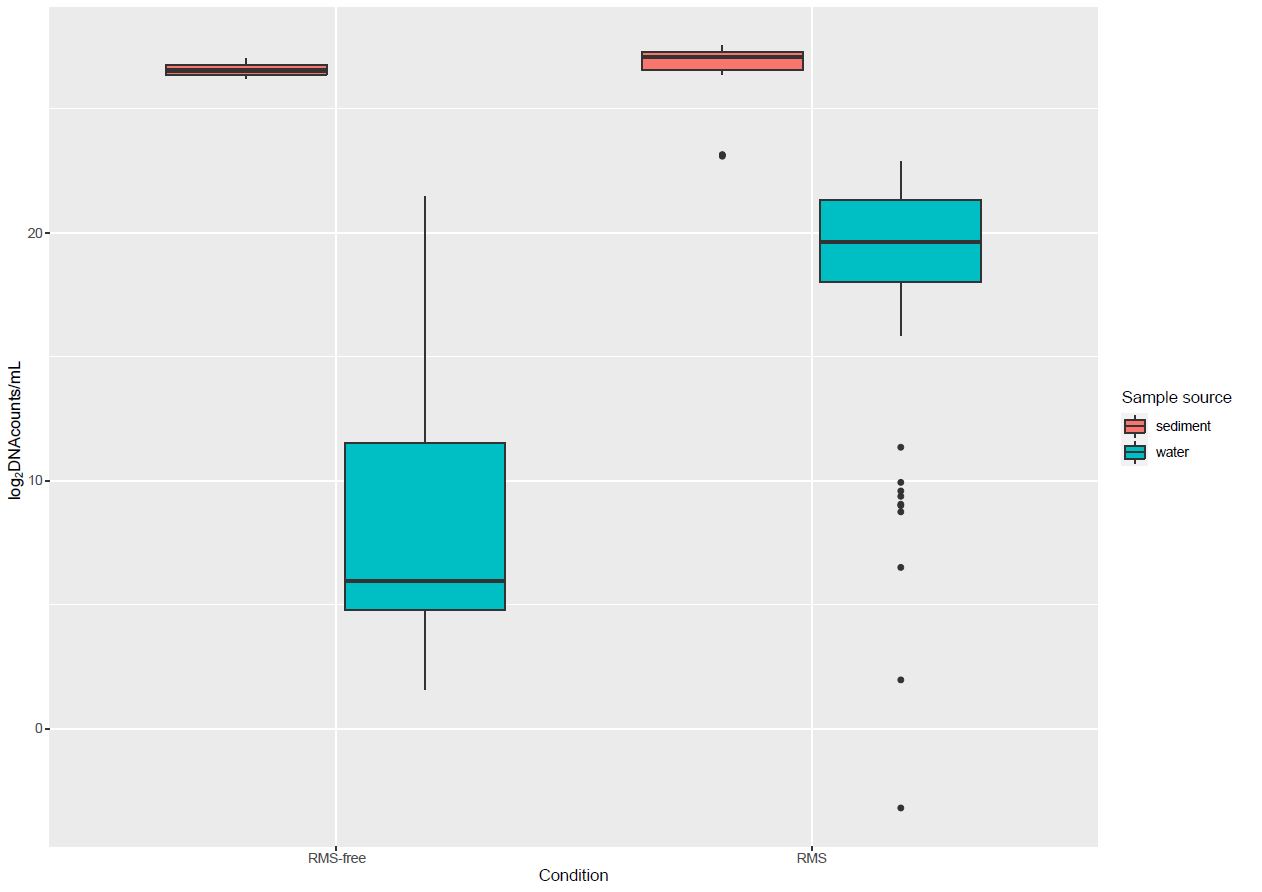


**Supplementary Figure 1.** 16S rDNA quantification of sediment and water samples, considering condition (RMS-free vs RMS) . Values are expressed as log2(DNA counts)/mL.


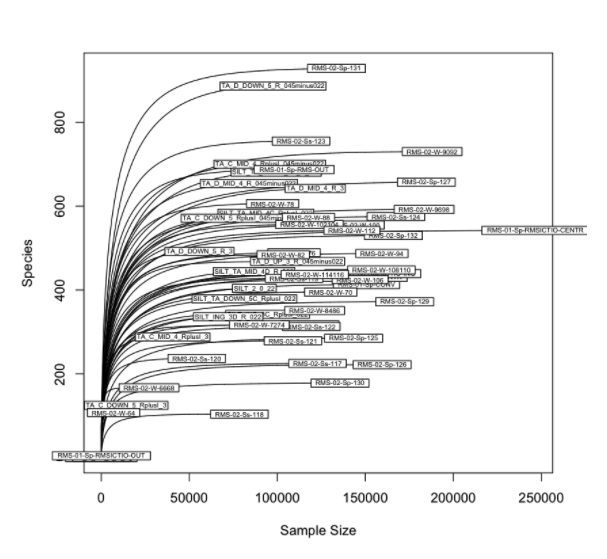


**Supplementary Figure** **2**. Rarefaction curves.


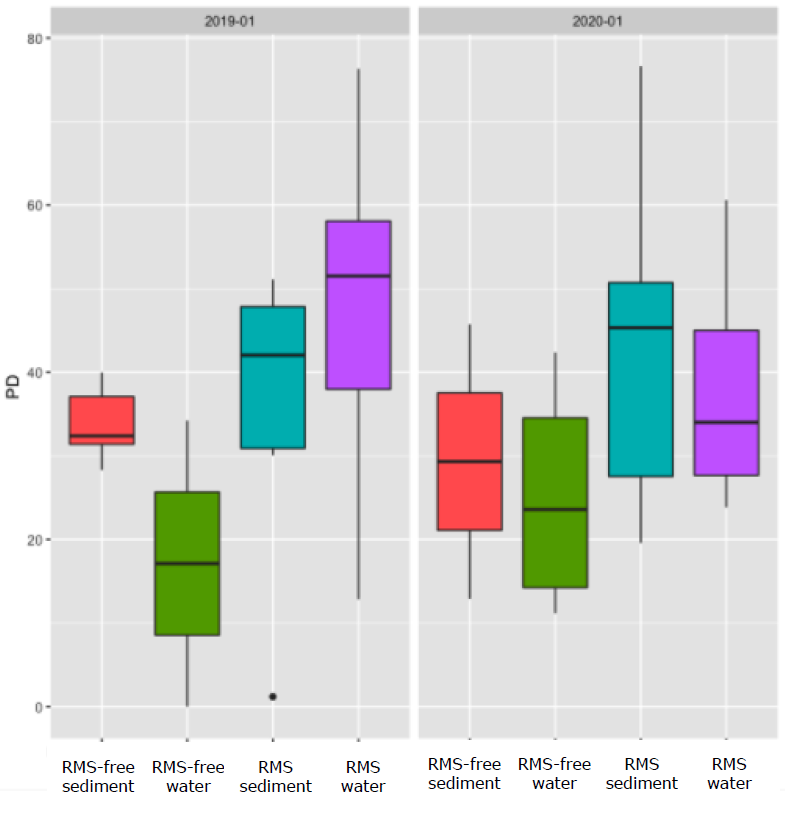


**Supplementary Figure** **3**. Alpha diversity based on Faith PD metric. The two sampling years are represented.

## Supplementary Tables

**Supplementary Table** **1**. Samples list.

| **#** | **Sampling_point** | **Condition** | **Sample_source** | **Condition_Source** | **Sampling_date** | **DNA extraction** | **Molecular assays performed** |
| --- | --- | --- | --- | --- | --- | --- | --- |
| 1 | p01 | RMS-free (no fish) | water | RMS-free_water | 2019-01 | yes | 16S qPCR  16S sequencing  RMS-MLO qPCR |
| 2 | p01 | RMS-free (no fish) | water | RMS-free_water | 2019-01 | yes |  |
| 3 | p01 | RMS-free (no fish) | water | RMS-free_water | 2019-01 | yes | 16S qPCR  16S sequencing  RMS-MLO qPCR |
| 4 | p02 | RMS-free (no fish) | sediment | RMS-free_sediment | 2019-01 | yes | 16S qPCR  16S sequencing  RMS-MLO qPCR |
| 5 | p02 | RMS-free (no fish) | sediment | RMS-free_sediment | 2019-01 | yes |  |
| 6 | p02 | RMS-free (no fish) | water | RMS-free_water | 2019-01 | yes | 16S qPCR  16S sequencing  RMS-MLO qPCR |
| 7 | p02 | RMS-free (no fish) | water | RMS-free_water | 2019-01 | yes |  |
| 8 | p02 | RMS-free (no fish) | water | RMS-free_water | 2019-01 | yes | 16S qPCR  16S sequencing  RMS-MLO qPCR |
| 9 | p03_C | RMS-free | sediment | RMS-free_sediment | 2019-01 | yes | 16S qPCR  16S sequencing  RMS-MLO qPCR |
| 10 | p03_C | RMS-free | sediment | RMS-free_sediment | 2019-01 | yes | 16S qPCR  16S sequencing  RMS-MLO qPCR |
| 11 | p03_C | RMS-free | water | RMS-free_water | 2019-01 | yes | 16S qPCR  16S sequencing  RMS-MLO qPCR |
| 12 | p03_C | RMS-free | water | RMS-free_water | 2019-01 | yes |  |
| 13 | p03_C | RMS-free | water | RMS-free_water | 2019-01 | yes | 16S qPCR  16S sequencing  RMS-MLO qPCR |
| 14 | p03_D | RMS-free | sediment | RMS-free_sediment | 2019-01 | yes | 16S qPCR  16S sequencing  RMS-MLO qPCR |
| 15 | p03_D | RMS-free | sediment | RMS-free_sediment | 2019-01 | NA | NA |
| 16 | p03_D | RMS-free | water | RMS-free_water | 2019-01 | yes | 16S qPCR  16S sequencing  RMS-MLO qPCR |
| 17 | p03_D | RMS-free | water | RMS-free_water | 2019-01 | yes |  |
| 18 | p03_D | RMS-free | water | RMS-free_water | 2019-01 | yes | 16S qPCR  16S sequencing  RMS-MLO qPCR |
| 19 | p04_C | RMS | sediment | RMS_sediment | 2019-01 | yes | 16S qPCR  16S sequencing  RMS-MLO qPCR |
| 20 | p04_C | RMS | sediment | RMS_sediment | 2019-01 | yes | 16S qPCR  16S sequencing  RMS-MLO qPCR |
| 21 | p04_C | RMS | water | RMS_water | 2019-01 | yes | 16S qPCR  16S sequencing  RMS-MLO qPCR |
| 22 | p04_C | RMS | water | RMS_water | 2019-01 | yes |  |
| 23 | p04_C | RMS | water | RMS_water | 2019-01 | yes | 16S qPCR  16S sequencing  RMS-MLO qPCR |
| 24 | p04_D | RMS | sediment | RMS_sediment | 2019-01 | yes | 16S qPCR  16S sequencing  RMS-MLO qPCR |
| 25 | p04_D | RMS | sediment | RMS_sediment | 2019-01 | yes | 16S qPCR  16S sequencing  RMS-MLO qPCR |
| 26 | p04_D | RMS | water | RMS_water | 2019-01 | yes | 16S qPCR  16S sequencing  RMS-MLO qPCR |
| 27 | p04_D | RMS | water | RMS_water | 2019-01 | yes |  |
| 28 | p04_D | RMS | water | RMS_water | 2019-01 | yes | 16S qPCR  16S sequencing  RMS-MLO qPCR |
| 29 | p05_C | RMS | sediment | RMS_sediment | 2019-01 | yes | 16S qPCR  16S sequencing  RMS-MLO qPCR |
| 30 | p05_C | RMS | sediment | RMS_sediment | 2019-01 | yes | 16S qPCR  16S sequencing  RMS-MLO qPCR |
| 31 | p05_C | RMS | water | RMS_water | 2019-01 | yes | 16S qPCR  16S sequencing  RMS-MLO qPCR |
| 32 | p05_C | RMS | water | RMS_water | 2019-01 | yes |  |
| 33 | p05_C | RMS | water | RMS_water | 2019-01 | yes | 16S qPCR  16S sequencing  RMS-MLO qPCR |
| 34 | p05_D | RMS | sediment | RMS_sediment | 2019-01 | yes | 16S qPCR  16S sequencing  RMS-MLO qPCR |
| 35 | p05_D | RMS | sediment | RMS_sediment | 2019-01 | yes | 16S qPCR  16S sequencing  RMS-MLO qPCR |
| 36 | p05_D | RMS | water | RMS_water | 2019-01 | yes | 16S qPCR  16S sequencing  RMS-MLO qPCR |
| 37 | p05_D | RMS | water | RMS_water | 2019-01 | yes |  |
| 38 | p05_D | RMS | water | RMS_water | 2019-01 | yes | 16S qPCR  16S sequencing  RMS-MLO qPCR |
| 39 | p01 | RMS-free (no fish) | water | RMS-free_water | 2020-01 | yes | 16S qPCR  16S sequencing  RMS-MLO qPCR |
| 40 | p01 | RMS-free (no fish) | water | RMS-free_water | 2020-01 | yes |  |
| 41 | p01 | RMS-free (no fish) | water | RMS-free_water | 2020-01 | yes | 16S qPCR  16S sequencing  RMS-MLO qPCR |
| 42 | p0102 | RMS | sediment | RMS_sediment | 2020-01 | yes | 16S qPCR  16S sequencing  RMS-MLO qPCR |
| 43 | p0102 | RMS | sediment | RMS_sediment | 2020-01 | yes | 16S qPCR  16S sequencing  RMS-MLO qPCR |
| 44 | p0102 | RMS | water | RMS_water | 2020-01 | yes | 16S qPCR  16S sequencing  RMS-MLO qPCR |
| 45 | p0102 | RMS | water | RMS_water | 2020-01 | yes |  |
| 46 | p0102 | RMS | water | RMS_water | 2020-01 | yes | 16S qPCR  16S sequencing  RMS-MLO qPCR |
| 47 | p02 | RMS-free (no fish) | sediment | RMS-free_sediment | 2020-01 | yes | 16S qPCR  16S sequencing  RMS-MLO qPCR |
| 48 | p02 | RMS-free (no fish) | sediment | RMS-free_sediment | 2020-01 | yes | 16S qPCR  16S sequencing  RMS-MLO qPCR |
| 49 | p02 | RMS-free (no fish) | water | RMS-free_water | 2020-01 | yes | 16S qPCR  16S sequencing  RMS-MLO qPCR |
| 50 | p02 | RMS-free (no fish) | water | RMS-free_water | 2020-01 | yes |  |
| 51 | p02 | RMS-free (no fish) | water | RMS-free_water | 2020-01 | yes | 16S qPCR  16S sequencing  RMS-MLO qPCR |
| 52 | p03_C | RMS | sediment | RMS_sediment | 2020-01 | yes | 16S qPCR  16S sequencing  RMS-MLO qPCR |
| 53 | p03_C | RMS | sediment | RMS_sediment | 2020-01 | yes | 16S qPCR  16S sequencing  RMS-MLO qPCR |
| 54 | p03_C | RMS | water | RMS_water | 2020-01 | yes | 16S qPCR  16S sequencing  RMS-MLO qPCR |
| 55 | p03_C | RMS | water | RMS_water | 2020-01 | yes |  |
| 56 | p03_C | RMS | water | RMS_water | 2020-01 | yes | 16S qPCR  16S sequencing  RMS-MLO qPCR |
| 57 | p03_D | RMS | sediment | RMS_sediment | 2020-01 | yes | 16S qPCR  16S sequencing  RMS-MLO qPCR |
| 58 | p03_D | RMS | sediment | RMS_sediment | 2020-01 | yes | 16S qPCR  16S sequencing  RMS-MLO qPCR |
| 59 | p03_D | RMS | water | RMS_water | 2020-01 | yes | 16S qPCR  16S sequencing  RMS-MLO qPCR |
| 60 | p03_D | RMS | water | RMS_water | 2020-01 | yes |  |
| 61 | p03_D | RMS | water | RMS_water | 2020-01 | yes | 16S qPCR  16S sequencing  RMS-MLO qPCR |
| 62 | p04_C | RMS | sediment | RMS_sediment | 2020-01 | yes | 16S qPCR  16S sequencing  RMS-MLO qPCR |
| 63 | p04_C | RMS | sediment | RMS_sediment | 2020-01 | yes | 16S qPCR  16S sequencing  RMS-MLO qPCR |
| 64 | p04_C | RMS | water | RMS_water | 2020-01 | yes | 16S qPCR  16S sequencing  RMS-MLO qPCR |
| 65 | p04_C | RMS | water | RMS_water | 2020-01 | yes |  |
| 66 | p04_C | RMS | water | RMS_water | 2020-01 | yes | 16S qPCR  16S sequencing  RMS-MLO qPCR |
| 67 | p04_D | RMS | sediment | RMS_sediment | 2020-01 | yes | 16S qPCR  16S sequencing  RMS-MLO qPCR |
| 68 | p04_D | RMS | sediment | RMS_sediment | 2020-01 | yes | 16S qPCR  16S sequencing  RMS-MLO qPCR |
| 69 | p04_D | RMS | water | RMS_water | 2020-01 | yes | 16S qPCR  16S sequencing  RMS-MLO qPCR |
| 70 | p04_D | RMS | water | RMS_water | 2020-01 | yes |  |
| 71 | p04_D | RMS | water | RMS_water | 2020-01 | yes | 16S qPCR  16S sequencing  RMS-MLO qPCR |
| 72 | p05_C | RMS | sediment | RMS_sediment | 2020-01 | yes | 16S qPCR  16S sequencing  RMS-MLO qPCR |
| 73 | p05_C | RMS | sediment | RMS_sediment | 2020-01 | yes | 16S qPCR  16S sequencing  RMS-MLO qPCR |
| 74 | p05_C | RMS | water | RMS_water | 2020-01 | yes | 16S qPCR  16S sequencing  RMS-MLO qPCR |
| 75 | p05_C | RMS | water | RMS_water | 2020-01 | yes | 16S qPCR  16S sequencing  RMS-MLO qPCR |
| 76 | p05_C | RMS | water | RMS_water | 2020-01 | yes | 16S qPCR  16S sequencing  RMS-MLO qPCR |
| 77 | p05_D | RMS | sediment | RMS_sediment | 2020-01 | yes | 16S qPCR  16S sequencing  RMS-MLO qPCR |
| 78 | p05_D | RMS | sediment | RMS_sediment | 2020-01 | yes | 16S qPCR  16S sequencing  RMS-MLO qPCR |
| 79 | p05_D | RMS | water | RMS_water | 2020-01 | yes | 16S qPCR  16S sequencing  RMS-MLO qPCR |
| 80 | p05_D | RMS | water | RMS_water | 2020-01 | yes | 16S qPCR  16S sequencing  RMS-MLO qPCR |
| 81 | p05_D | RMS | water | RMS_water | 2020-01 | yes | 16S qPCR  16S sequencing  RMS-MLO qPCR |
| 82 | Blank | Blank | Blank | Blank | Blank | yes | 16S qPCR  16S sequencing  RMS-MLO qPCR |
| 83 | Blank | Blank | Blank | Blank | Blank | yes | 16S qPCR  16S sequencing  RMS-MLO qPCR |
| 84 | Blank | Blank | Blank | Blank | Blank | yes | 16S qPCR  16S sequencing  RMS-MLO qPCR |
| 85 | Blank | Blank | Blank | Blank | Blank | yes | 16S qPCR  16S sequencing  RMS-MLO qPCR |

**Supplementary Table** **2**. Sample list with the number of features obtained after 16S rRNA Illumina sequencing.

In red, samples excluded from further analyses are indicated.

| **Sampling_point** | **Condition** | **Sample_source** | **Condition_Source** | **Sampling_date** | **Features** |
| --- | --- | --- | --- | --- | --- |
| p02 | RMS-free (no fish) | sediment | RMS-free_sediment | 2019-01 | 150654 |
| p02 | RMS-free (no fish) | sediment | RMS-free_sediment | 2019-01 | 86956 |
| p02 | RMS-free (no fish) | water | RMS-free_water | 2019-01 | 0 |
| p03_C | RMS-free | sediment | RMS-free_sediment | 2019-01 | 154237 |
| p03_C | RMS-free | sediment | RMS-free_sediment | 2019-01 | 94490 |
| p03_C | RMS-free | water | RMS-free_water | 2019-01 | 1 |
| p03_D | RMS-free | sediment | RMS-free_sediment | 2019-01 | 72018 |
| p03_D | RMS-free | water | RMS-free_water | 2019-01 | 111460 |
| p03_D | RMS-free | water | RMS-free_water | 2019-01 | 1 |
| p04_C | RMS | sediment | RMS_sediment | 2019-01 | 246606 |
| p04_C | RMS | sediment | RMS_sediment | 2019-01 | 93471 |
| p04_C | RMS | water | RMS_water | 2019-01 | 95658 |
| p04_C | RMS | water | RMS_water | 2019-01 | 40503 |
| p04_D | RMS | sediment | RMS_sediment | 2019-01 | 148492 |
| p04_D | RMS | sediment | RMS_sediment | 2019-01 | 86819 |
| p04_D | RMS | water | RMS_water | 2019-01 | 83888 |
| p04_D | RMS | water | RMS_water | 2019-01 | 121529 |
| p05_C | RMS | sediment | RMS_sediment | 2019-01 | 49 |
| p05_C | RMS | sediment | RMS_sediment | 2019-01 | 81295 |
| p05_C | RMS | water | RMS_water | 2019-01 | 79547 |
| p05_C | RMS | water | RMS_water | 2019-01 | 14122 |
| p05_D | RMS | sediment | RMS_sediment | 2019-01 | 109548 |
| p05_D | RMS | sediment | RMS_sediment | 2019-01 | 99307 |
| p05_D | RMS | water | RMS_water | 2019-01 | 97547 |
| p05_D | RMS | water | RMS_water | 2019-01 | 55799 |
| p01 | RMS-free (no fish) | water | RMS-free_water | 2020-01 | 6960 |
| p01 | RMS-free (no fish) | water | RMS-free_water | 2020-01 | 27097 |
| p0102 | RMS | sediment | RMS_sediment | 2020-01 | 143355 |
| p0102 | RMS | sediment | RMS_sediment | 2020-01 | 122830 |
| p0102 | RMS | water | RMS_water | 2020-01 | 130332 |
| p0102 | RMS | water | RMS_water | 2020-01 | 89801 |
| p02 | RMS-free (no fish) | sediment | RMS-free_sediment | 2020-01 | 159514 |
| p02 | RMS-free (no fish) | sediment | RMS-free_sediment | 2020-01 | 78465 |
| p02 | RMS-free (no fish) | water | RMS-free_water | 2020-01 | 109465 |
| p02 | RMS-free (no fish) | water | RMS-free_water | 2020-01 | 97328 |
| p03_C | RMS | sediment | RMS_sediment | 2020-01 | 184642 |
| p03_C | RMS | sediment | RMS_sediment | 2020-01 | 109506 |
| p03_C | RMS | water | RMS_water | 2020-01 | 103337 |
| p03_C | RMS | water | RMS_water | 2020-01 | 121176 |
| p03_D | RMS | sediment | RMS_sediment | 2020-01 | 118535 |
| p03_D | RMS | sediment | RMS_sediment | 2020-01 | 54228 |
| p03_D | RMS | water | RMS_water | 2020-01 | 117701 |
| p03_D | RMS | water | RMS_water | 2020-01 | 187957 |
| p04_C | RMS | sediment | RMS_sediment | 2020-01 | 172367 |
| p04_C | RMS | sediment | RMS_sediment | 2020-01 | 108862 |
| p04_C | RMS | water | RMS_water | 2020-01 | 159401 |
| p04_C | RMS | water | RMS_water | 2020-01 | 183383 |
| p04_D | RMS | sediment | RMS_sediment | 2020-01 | 135593 |
| p04_D | RMS | sediment | RMS_sediment | 2020-01 | 119288 |
| p04_D | RMS | water | RMS_water | 2020-01 | 144861 |
| p04_D | RMS | water | RMS_water | 2020-01 | 118228 |
| p05_C | RMS | sediment | RMS_sediment | 2020-01 | 133579 |
| p05_C | RMS | sediment | RMS_sediment | 2020-01 | 113491 |
| p05_C | RMS | water | RMS_water | 2020-01 | 147026 |
| p05_C | RMS | water | RMS_water | 2020-01 | 159147 |
| p05_D | RMS | sediment | RMS_sediment | 2020-01 | 165940 |
| p05_D | RMS | sediment | RMS_sediment | 2020-01 | 167363 |
| p05_D | RMS | water | RMS_water | 2020-01 | 142355 |
| p05_D | RMS | water | RMS_water | 2020-01 | 121281 |

**Supplementary Table** **3**. 16S qPCR results

| **Sampling_point** | **Condition** | **Sample_source** | **Condition_Source** | **Sampling_date** | **log2DNAcounts/mL** |
| --- | --- | --- | --- | --- | --- |
| p01 | RMS-free (no fish) | water | RMS-free_water | 2019-01 | 2.71 |
| p01 | RMS-free (no fish) | water | RMS-free_water | 2019-01 | 2.24 |
| p01 | RMS-free (no fish) | water | RMS-free_water | 2019-01 | 2.30 |
| p01 | RMS-free (no fish) | water | RMS-free_water | 2019-01 | 2.38 |
| p01 | RMS-free (no fish) | water | RMS-free_water | 2019-01 | 4.24 |
| p01 | RMS-free (no fish) | water | RMS-free_water | 2019-01 | 2.12 |
| p01 | RMS-free (no fish) | water | RMS-free_water | 2019-01 | 5.59 |
| p01 | RMS-free (no fish) | water | RMS-free_water | 2019-01 | 5.67 |
| p01 | RMS-free (no fish) | water | RMS-free_water | 2019-01 | 6.35 |
| p02 | RMS-free (no fish) | sediment | RMS-free_sediment | 2019-01 | 26.67 |
| p02 | RMS-free (no fish) | sediment | RMS-free_sediment | 2019-01 | 26.71 |
| p02 | RMS-free (no fish) | sediment | RMS-free_sediment | 2019-01 | 26.73 |
| p02 | RMS-free (no fish) | water | RMS-free_water | 2019-01 | 2.89 |
| p02 | RMS-free (no fish) | water | RMS-free_water | 2019-01 | 4.28 |
| p02 | RMS-free (no fish) | water | RMS-free_water | 2019-01 | 4.49 |
| p02 | RMS-free (no fish) | water | RMS-free_water | 2019-01 | 4.04 |
| p02 | RMS-free (no fish) | water | RMS-free_water | 2019-01 | 4.29 |
| p02 | RMS-free (no fish) | water | RMS-free_water | 2019-01 | 1.57 |
| p02 | RMS-free (no fish) | water | RMS-free_water | 2019-01 | 11.47 |
| p02 | RMS-free (no fish) | water | RMS-free_water | 2019-01 | 11.57 |
| p02 | RMS-free (no fish) | water | RMS-free_water | 2019-01 | 11.56 |
| p03_C | RMS-free | sediment | RMS-free_sediment | 2019-01 | 26.41 |
| p03_C | RMS-free | sediment | RMS-free_sediment | 2019-01 | 26.41 |
| p03_C | RMS-free | sediment | RMS-free_sediment | 2019-01 | 26.42 |
| p03_C | RMS-free | water | RMS-free_water | 2019-01 | 5.73 |
| p03_C | RMS-free | water | RMS-free_water | 2019-01 | 5.17 |
| p03_C | RMS-free | water | RMS-free_water | 2019-01 | 5.57 |
| p03_C | RMS-free | water | RMS-free_water | 2019-01 | 5.77 |
| p03_C | RMS-free | water | RMS-free_water | 2019-01 | 5.46 |
| p03_C | RMS-free | water | RMS-free_water | 2019-01 | 6.16 |
| p03_C | RMS-free | water | RMS-free_water | 2019-01 | 4.81 |
| p03_C | RMS-free | water | RMS-free_water | 2019-01 | 5.22 |
| p03_C | RMS-free | water | RMS-free_water | 2019-01 | 5.25 |
| p03_D | RMS-free | sediment | RMS-free_sediment | 2019-01 | 26.26 |
| p03_D | RMS-free | sediment | RMS-free_sediment | 2019-01 | 26.24 |
| p03_D | RMS-free | sediment | RMS-free_sediment | 2019-01 | 26.26 |
| p03_D | RMS-free | water | RMS-free_water | 2019-01 | 4.68 |
| p03_D | RMS-free | water | RMS-free_water | 2019-01 | 5.52 |
| p03_D | RMS-free | water | RMS-free_water | 2019-01 | 5.61 |
| p03_D | RMS-free | water | RMS-free_water | 2019-01 | 18.27 |
| p03_D | RMS-free | water | RMS-free_water | 2019-01 | 18.19 |
| p03_D | RMS-free | water | RMS-free_water | 2019-01 | 18.24 |
| p03_D | RMS-free | water | RMS-free_water | 2019-01 | 6.34 |
| p03_D | RMS-free | water | RMS-free_water | 2019-01 | 5.60 |
| p03_D | RMS-free | water | RMS-free_water | 2019-01 | 4.78 |
| p04_C | RMS | sediment | RMS_sediment | 2019-01 | 23.14 |
| p04_C | RMS | sediment | RMS_sediment | 2019-01 | 23.09 |
| p04_C | RMS | sediment | RMS_sediment | 2019-01 | 23.15 |
| p04_C | RMS | water | RMS_water | 2019-01 | 21.96 |
| p04_C | RMS | water | RMS_water | 2019-01 | 22.26 |
| p04_C | RMS | water | RMS_water | 2019-01 | 22.23 |
| p04_C | RMS | water | RMS_water | 2019-01 | 19.59 |
| p04_C | RMS | water | RMS_water | 2019-01 | 19.63 |
| p04_C | RMS | water | RMS_water | 2019-01 | 19.65 |
| p04_C | RMS | water | RMS_water | 2019-01 | 21.91 |
| p04_C | RMS | water | RMS_water | 2019-01 | 21.94 |
| p04_C | RMS | water | RMS_water | 2019-01 | 21.96 |
| p04_D | RMS | sediment | RMS_sediment | 2019-01 | 26.60 |
| p04_D | RMS | sediment | RMS_sediment | 2019-01 | 26.60 |
| p04_D | RMS | sediment | RMS_sediment | 2019-01 | 26.60 |
| p04_D | RMS | water | RMS_water | 2019-01 | 18.00 |
| p04_D | RMS | water | RMS_water | 2019-01 | 18.00 |
| p04_D | RMS | water | RMS_water | 2019-01 | 18.00 |
| p04_D | RMS | water | RMS_water | 2019-01 | 18.00 |
| p04_D | RMS | water | RMS_water | 2019-01 | 18.00 |
| p04_D | RMS | water | RMS_water | 2019-01 | 18.00 |
| p04_D | RMS | water | RMS_water | 2019-01 | 18.00 |
| p04_D | RMS | water | RMS_water | 2019-01 | 18.00 |
| p04_D | RMS | water | RMS_water | 2019-01 | 18.00 |
| p05_C | RMS | sediment | RMS_sediment | 2019-01 | 26.51 |
| p05_C | RMS | sediment | RMS_sediment | 2019-01 | 26.55 |
| p05_C | RMS | sediment | RMS_sediment | 2019-01 | 26.53 |
| p05_C | RMS | water | RMS_water | 2019-01 | 20.21 |
| p05_C | RMS | water | RMS_water | 2019-01 | 20.19 |
| p05_C | RMS | water | RMS_water | 2019-01 | 20.26 |
| p05_C | RMS | water | RMS_water | 2019-01 | 17.06 |
| p05_C | RMS | water | RMS_water | 2019-01 | 16.35 |
| p05_C | RMS | water | RMS_water | 2019-01 | 15.84 |
| p05_C | RMS | water | RMS_water | 2019-01 | 22.31 |
| p05_C | RMS | water | RMS_water | 2019-01 | 22.31 |
| p05_C | RMS | water | RMS_water | 2019-01 | 22.36 |
| p05_D | RMS | sediment | RMS_sediment | 2019-01 | 26.39 |
| p05_D | RMS | sediment | RMS_sediment | 2019-01 | 26.42 |
| p05_D | RMS | sediment | RMS_sediment | 2019-01 | 26.45 |
| p05_D | RMS | water | RMS_water | 2019-01 | 22.83 |
| p05_D | RMS | water | RMS_water | 2019-01 | 22.86 |
| p05_D | RMS | water | RMS_water | 2019-01 | 22.89 |
| p05_D | RMS | water | RMS_water | 2019-01 | 18.00 |
| p05_D | RMS | water | RMS_water | 2019-01 | 18.00 |
| p05_D | RMS | water | RMS_water | 2019-01 | 18.00 |
| p05_D | RMS | water | RMS_water | 2019-01 | 18.00 |
| p05_D | RMS | water | RMS_water | 2019-01 | 18.00 |
| p05_D | RMS | water | RMS_water | 2019-01 | 18.00 |
| p01 | RMS-free (no fish) | water | RMS-free_water | 2020-01 | 10.08 |
| p01 | RMS-free (no fish) | water | RMS-free_water | 2020-01 | 9.65 |
| p01 | RMS-free (no fish) | water | RMS-free_water | 2020-01 | 9.92 |
| p01 | RMS-free (no fish) | water | RMS-free_water | 2020-01 | 12.84 |
| p01 | RMS-free (no fish) | water | RMS-free_water | 2020-01 | 12.74 |
| p01 | RMS-free (no fish) | water | RMS-free_water | 2020-01 | 12.72 |
| p01 | RMS-free (no fish) | water | RMS-free_water | 2020-01 | 10.05 |
| p01 | RMS-free (no fish) | water | RMS-free_water | 2020-01 | 10.28 |
| p01 | RMS-free (no fish) | water | RMS-free_water | 2020-01 | 10.28 |
| p0102 | RMS | sediment | RMS_sediment | 2020-01 | 27.36 |
| p0102 | RMS | sediment | RMS_sediment | 2020-01 | 27.30 |
| p0102 | RMS | sediment | RMS_sediment | 2020-01 | 27.33 |
| p0102 | RMS | water | RMS_water | 2020-01 | 21.35 |
| p0102 | RMS | water | RMS_water | 2020-01 | 21.33 |
| p0102 | RMS | water | RMS_water | 2020-01 | 21.34 |
| p0102 | RMS | water | RMS_water | 2020-01 | 20.93 |
| p0102 | RMS | water | RMS_water | 2020-01 | 20.88 |
| p0102 | RMS | water | RMS_water | 2020-01 | 20.84 |
| p0102 | RMS | water | RMS_water | 2020-01 | 19.59 |
| p0102 | RMS | water | RMS_water | 2020-01 | 19.75 |
| p0102 | RMS | water | RMS_water | 2020-01 | 19.60 |
| p02 | RMS-free (no fish) | sediment | RMS-free_sediment | 2020-01 | 27.03 |
| p02 | RMS-free (no fish) | sediment | RMS-free_sediment | 2020-01 | 26.95 |
| p02 | RMS-free (no fish) | sediment | RMS-free_sediment | 2020-01 | 27.03 |
| p02 | RMS-free (no fish) | water | RMS-free_water | 2020-01 | 21.35 |
| p02 | RMS-free (no fish) | water | RMS-free_water | 2020-01 | 21.20 |
| p02 | RMS-free (no fish) | water | RMS-free_water | 2020-01 | 21.27 |
| p02 | RMS-free (no fish) | water | RMS-free_water | 2020-01 | 21.46 |
| p02 | RMS-free (no fish) | water | RMS-free_water | 2020-01 | 21.46 |
| p02 | RMS-free (no fish) | water | RMS-free_water | 2020-01 | 21.42 |
| p02 | RMS-free (no fish) | water | RMS-free_water | 2020-01 | 7.84 |
| p02 | RMS-free (no fish) | water | RMS-free_water | 2020-01 | 7.78 |
| p02 | RMS-free (no fish) | water | RMS-free_water | 2020-01 | 7.74 |
| p03_C | RMS | sediment | RMS_sediment | 2020-01 | 27.29 |
| p03_C | RMS | sediment | RMS_sediment | 2020-01 | 27.31 |
| p03_C | RMS | sediment | RMS_sediment | 2020-01 | 27.31 |
| p03_C | RMS | water | RMS_water | 2020-01 | 21.41 |
| p03_C | RMS | water | RMS_water | 2020-01 | 21.39 |
| p03_C | RMS | water | RMS_water | 2020-01 | 21.36 |
| p03_C | RMS | water | RMS_water | 2020-01 | 21.31 |
| p03_C | RMS | water | RMS_water | 2020-01 | 21.42 |
| p03_C | RMS | water | RMS_water | 2020-01 | 21.34 |
| p03_C | RMS | water | RMS_water | 2020-01 | 8.99 |
| p03_C | RMS | water | RMS_water | 2020-01 | 9.05 |
| p03_C | RMS | water | RMS_water | 2020-01 | 8.74 |
| p03_D | RMS | sediment | RMS_sediment | 2020-01 | 27.13 |
| p03_D | RMS | sediment | RMS_sediment | 2020-01 | 27.06 |
| p03_D | RMS | sediment | RMS_sediment | 2020-01 | 27.09 |
| p03_D | RMS | water | RMS_water | 2020-01 | 19.46 |
| p03_D | RMS | water | RMS_water | 2020-01 | 20.04 |
| p03_D | RMS | water | RMS_water | 2020-01 | 20.00 |
| p03_D | RMS | water | RMS_water | 2020-01 | 19.38 |
| p03_D | RMS | water | RMS_water | 2020-01 | 19.23 |
| p03_D | RMS | water | RMS_water | 2020-01 | 18.99 |
| p03_D | RMS | water | RMS_water | 2020-01 | 19.71 |
| p03_D | RMS | water | RMS_water | 2020-01 | 19.62 |
| p03_D | RMS | water | RMS_water | 2020-01 | 19.64 |
| p04_C | RMS | sediment | RMS_sediment | 2020-01 | 27.53 |
| p04_C | RMS | sediment | RMS_sediment | 2020-01 | 27.55 |
| p04_C | RMS | sediment | RMS_sediment | 2020-01 | 27.55 |
| p04_C | RMS | water | RMS_water | 2020-01 | 11.35 |
| p04_C | RMS | water | RMS_water | 2020-01 | 6.50 |
| p04_C | RMS | water | RMS_water | 2020-01 | 1.96 |
| p04_C | RMS | water | RMS_water | 2020-01 | 20.26 |
| p04_C | RMS | water | RMS_water | 2020-01 | 20.23 |
| p04_C | RMS | water | RMS_water | 2020-01 | 20.43 |
| p04_C | RMS | water | RMS_water | 2020-01 | 19.05 |
| p04_C | RMS | water | RMS_water | 2020-01 | 19.22 |
| p04_C | RMS | water | RMS_water | 2020-01 | 19.12 |
| p04_D | RMS | sediment | RMS_sediment | 2020-01 | 27.38 |
| p04_D | RMS | sediment | RMS_sediment | 2020-01 | 27.38 |
| p04_D | RMS | sediment | RMS_sediment | 2020-01 | 27.38 |
| p04_D | RMS | water | RMS_water | 2020-01 | 21.47 |
| p04_D | RMS | water | RMS_water | 2020-01 | 21.33 |
| p04_D | RMS | water | RMS_water | 2020-01 | 21.40 |
| p04_D | RMS | water | RMS_water | 2020-01 | 19.49 |
| p04_D | RMS | water | RMS_water | 2020-01 | 19.49 |
| p04_D | RMS | water | RMS_water | 2020-01 | 19.48 |
| p04_D | RMS | water | RMS_water | 2020-01 | 19.01 |
| p04_D | RMS | water | RMS_water | 2020-01 | 18.88 |
| p04_D | RMS | water | RMS_water | 2020-01 | 18.91 |
| p05_C | RMS | sediment | RMS_sediment | 2020-01 | 27.29 |
| p05_C | RMS | sediment | RMS_sediment | 2020-01 | 27.29 |
| p05_C | RMS | sediment | RMS_sediment | 2020-01 | 27.28 |
| p05_C | RMS | water | RMS_water | 2020-01 | 21.17 |
| p05_C | RMS | water | RMS_water | 2020-01 | 20.79 |
| p05_C | RMS | water | RMS_water | 2020-01 | 20.58 |
| p05_C | RMS | water | RMS_water | 2020-01 | 19.73 |
| p05_C | RMS | water | RMS_water | 2020-01 | 19.89 |
| p05_C | RMS | water | RMS_water | 2020-01 | 19.85 |
| p05_C | RMS | water | RMS_water | 2020-01 | 20.39 |
| p05_C | RMS | water | RMS_water | 2020-01 | 20.41 |
| p05_C | RMS | water | RMS_water | 2020-01 | -3.21 |
| p05_D | RMS | sediment | RMS_sediment | 2020-01 | 26.95 |
| p05_D | RMS | sediment | RMS_sediment | 2020-01 | 26.96 |
| p05_D | RMS | sediment | RMS_sediment | 2020-01 | 26.97 |
| p05_D | RMS | water | RMS_water | 2020-01 | 19.25 |
| p05_D | RMS | water | RMS_water | 2020-01 | 19.48 |
| p05_D | RMS | water | RMS_water | 2020-01 | 18.71 |
| p05_D | RMS | water | RMS_water | 2020-01 | 21.59 |
| p05_D | RMS | water | RMS_water | 2020-01 | 21.59 |
| p05_D | RMS | water | RMS_water | 2020-01 | 21.62 |
| p05_D | RMS | water | RMS_water | 2020-01 | 9.93 |
| p05_D | RMS | water | RMS_water | 2020-01 | 9.37 |
| p05_D | RMS | water | RMS_water | 2020-01 | 9.58 |
| Blank | NA | water | NA | NA | Undetected |
| Blank | NA | water | NA | NA | Undetected |
| Blank | NA | sediment | NA | NA | Undetected |
| Blank | NA | sediment | NA | NA | Undetected |

**Supplementary Table** **4**. Kruskal–Wallis H test.

|  | **Result** |
| --- | --- |
| **H** | 5.604014168 |
| **p-value** | 0.06068813413 |

**Supplementary Table** **5**. Pairwise Kruskal–Wallis H test for Source_Condition.

| **Group 1** | **Group 2** | **H** | **p-value** | **q-value** |
| --- | --- | --- | --- | --- |
| **RMS_sediment (n=21)** | **RMS_water (n=22)** | 0.399055 | 0.527577 | 0.586197 |
|  | **RMS-free_sediment (n=7)** | 6.351161 | 0.01173 | 0.117304 |
|  | **RMS-free_water (n=4)** | 0.664835 | 0.414858 | 0.518573 |
| **RMS_water (n=22)** | **RMS-free_sediment (n=7)** | 4.155844 | 0.041491 | 0.207455 |
|  | **RMS-free_water (n=4)** | 0.080808 | 0.776205 | 0.776205 |
| **RMS-free_sediment (n=7)** | **RMS-free_water (n=4)** | 1.75 | 0.185877 | 0.265538 |

**Supplementary Table** **6**. Kruskal–Wallis H test for sampling years.

| **Group 1** | **Group 2** | **H** | **p-value** | **q-value** |
| --- | --- | --- | --- | --- |
| **2019-01 (n=21)** | **2020-01 (n=33)** | 2.81157 | 0.093587 | 0.097918 |

**Supplementary Table** **7**. Permanova results

| **Combination** | **Df** | **SumsOfSqs** | **MeanSqs** | **F.Model** | **R2** | **Pr(>F)** |
| --- | --- | --- | --- | --- | --- | --- |
| **Condition_Source** | 3 | 4.7786 | 1.59287 | 17.5846 | **0.43660** | 9.999e-05 |
| **Sampling_Date** | 1 | 0.9360 | 0.93601 | 10.3331 | 0.08552 | 9.999e-05 |
| **Condition_Source:Sampling_date** | 3 | 1.0637 | 0.35457 | 3.9143 | 0.09719 | 9.999e-05 |
| **Residuals** | 46 | 4.1668 | 0.09058 |  | 0.38070 |  |
| **Total** | 53 | 10.9452 |  |  | 1.00000 |  |

**Supplementary Table** **8**. Permanova results for Source_Condition.

| **Combination** | **SumsOfSqs** | **MeanSqs** | **F.Model** | **R2** | **p-value** | **p-value corrected** |
| --- | --- | --- | --- | --- | --- | --- |
| **RMS-free_sediment <-> RMS-free_water** | 0.739441 | 0.739441 | 3.59031 | 0.28516453 | 0.026973027 | 0.04045954 |
| **RMS-free_sediment <-> RMS_sediment** | 0.3200323 | 0.3200323 | 2.089958 | 0.07440232 | 0.073926074 | 0.088711289 |
| **RMS-free_sediment <-> RMS_water** | 1.8145471 | 1.8145471 | 15.819756 | 0.36944994 | 0.000999001 | 0.001998002 |
| **RMS-free_water <-> RMS_sediment** | 1.0350285 | 1.0350285 | 7.755261 | 0.25216047 | 0.000999001 | 0.001998002 |
| RMS-free_water <-> RMS_water | 0.122142 | 0.122142 | 1.341478 | 0.05293606 | 0.250749251 | 0.250749251 |
| **RMS_sediment <-> RMS_water** | 3.7041186 | 3.7041186 | 35.212229 | 0.46202859 | 0.000999001 | 0.001998002 |

**Supplementary Table 9**. Results of the molecular analyses for RMS-MLO presence.

| **#** | **Sampling_point** | **Condition** | **Sample_source** | **Condition_Source** | **Sampling_date** | **RMS-MLO qPCR** |
| --- | --- | --- | --- | --- | --- | --- |
| 1 | p01 | RMS-free (no fish) | water | RMS-free_water | 2019-01 | NEG |
| 2 | p01 | RMS-free (no fish) | water | RMS-free_water | 2019-01 | NEG |
| 3 | p02 | RMS-free (no fish) | sediment | RMS-free_sediment | 2019-01 | **POS** |
| 4 | p02 | RMS-free (no fish) | sediment | RMS-free_sediment | 2019-01 | **POS** |
| 5 | p02 | RMS-free (no fish) | water | RMS-free_water | 2019-01 | NEG |
| 6 | p02 | RMS-free (no fish) | water | RMS-free_water | 2019-01 | NEG |
| 7 | p03_C | RMS-free | sediment | RMS-free_sediment | 2019-01 | **POS** |
| 8 | p03_C | RMS-free | sediment | RMS-free_sediment | 2019-01 | **POS** |
| 9 | p03_C | RMS-free | water | RMS-free_water | 2019-01 | NEG |
| 10 | p03_C | RMS-free | water | RMS-free_water | 2019-01 | NEG |
| 11 | p03_D | RMS-free | sediment | RMS-free_sediment | 2019-01 | **POS** |
| 12 | p03_D | RMS-free | water | RMS-free_water | 2019-01 | NEG |
| 13 | p03_D | RMS-free | water | RMS-free_water | 2019-01 | NEG |
| 14 | p04_C | RMS | sediment | RMS_sediment | 2019-01 | **POS** |
| 15 | p04_C | RMS | sediment | RMS_sediment | 2019-01 | **POS** |
| 16 | p04_C | RMS | water | RMS_water | 2019-01 | **POS** |
| 17 | p04_C | RMS | water | RMS_water | 2019-01 | **POS** |
| 18 | p04_D | RMS | sediment | RMS_sediment | 2019-01 | **POS** |
| 19 | p04_D | RMS | sediment | RMS_sediment | 2019-01 | **POS** |
| 20 | p04_D | RMS | water | RMS_water | 2019-01 | NEG |
| 21 | p04_D | RMS | water | RMS_water | 2019-01 | **POS** |
| 22 | p05_C | RMS | sediment | RMS_sediment | 2019-01 | **POS** |
| 23 | p05_C | RMS | sediment | RMS_sediment | 2019-01 | **POS** |
| 24 | p05_C | RMS | water | RMS_water | 2019-01 | NEG |
| 25 | p05_C | RMS | water | RMS_water | 2019-01 | **POS** |
| 26 | p05_D | RMS | sediment | RMS_sediment | 2019-01 | **POS** |
| 27 | p05_D | RMS | sediment | RMS_sediment | 2019-01 | **POS** |
| 28 | p05_D | RMS | water | RMS_water | 2019-01 | **POS** |
| 29 | p05_D | RMS | water | RMS_water | 2019-01 | **POS** |
| 30 | p01 | RMS-free (no fish) | water | RMS-free_water | 2020-01 | NEG |
| 31 | p01 | RMS-free (no fish) | water | RMS-free_water | 2020-01 | NEG |
| 32 | p0102 | RMS | sediment | RMS_sediment | 2020-01 | **POS** |
| 33 | p0102 | RMS | sediment | RMS_sediment | 2020-01 | **POS** |
| 34 | p0102 | RMS | water | RMS_water | 2020-01 | NEG |
| 35 | p0102 | RMS | water | RMS_water | 2020-01 | **POS** |
| 36 | p02 | RMS-free (no fish) | sediment | RMS-free_sediment | 2020-01 | NEG |
| 37 | p02 | RMS-free (no fish) | sediment | RMS-free_sediment | 2020-01 | NEG |
| 38 | p02 | RMS-free (no fish) | water | RMS-free_water | 2020-01 | **POS** |
| 39 | p02 | RMS-free (no fish) | water | RMS-free_water | 2020-01 | **POS** |
| 40 | p03_C | RMS | sediment | RMS_sediment | 2020-01 | NEG |
| 41 | p03_C | RMS | sediment | RMS_sediment | 2020-01 | NEG |
| 42 | p03_C | RMS | water | RMS_water | 2020-01 | **POS** |
| 43 | p03_C | RMS | water | RMS_water | 2020-01 | **POS** |
| 44 | p03_D | RMS | sediment | RMS_sediment | 2020-01 | **POS** |
| 45 | p03_D | RMS | sediment | RMS_sediment | 2020-01 | **POS** |
| 46 | p03_D | RMS | water | RMS_water | 2020-01 | **POS** |
| 47 | p03_D | RMS | water | RMS_water | 2020-01 | NEG |
| 48 | p04_C | RMS | sediment | RMS_sediment | 2020-01 | **POS** |
| 49 | p04_C | RMS | sediment | RMS_sediment | 2020-01 | **POS** |
| 50 | p04_C | RMS | water | RMS_water | 2020-01 | **POS** |
| 51 | p04_C | RMS | water | RMS_water | 2020-01 | NEG |
| 52 | p04_D | RMS | sediment | RMS_sediment | 2020-01 | NEG |
| 53 | p04_D | RMS | sediment | RMS_sediment | 2020-01 | NEG |
| 54 | p04_D | RMS | water | RMS_water | 2020-01 | **POS** |
| 55 | p04_D | RMS | water | RMS_water | 2020-01 | NEG |
| 56 | p05_C | RMS | sediment | RMS_sediment | 2020-01 | **POS** |
| 57 | p05_C | RMS | sediment | RMS_sediment | 2020-01 | **POS** |
| 58 | p05_C | RMS | water | RMS_water | 2020-01 | NEG |
| 59 | p05_C | RMS | water | RMS_water | 2020-01 | NEG |
| 60 | p05_D | RMS | sediment | RMS_sediment | 2020-01 | **POS** |
| 61 | p05_D | RMS | sediment | RMS_sediment | 2020-01 | **POS** |
| 62 | p05_D | RMS | water | RMS_water | 2020-01 | **POS** |
| 63 | p05_D | RMS | water | RMS_water | 2020-01 | NEG |
| 64 | Blank | NA | water | NA | NA | NEG |
| 65 | Blank | NA | water | NA | NA | NEG |
| 66 | Blank | NA | sediment | NA | NA | NEG |
| 67 | Blank | NA | sediment | NA | NA | NEG |
